# Enhanced oral absorption of insulin: hydrophobic ion pairing and a self-microemulsifying drug delivery system using a D-optimal mixture design

**DOI:** 10.1080/10717544.2022.2118399

**Published:** 2022-09-01

**Authors:** Yoon Tae Goo, Sangkil Lee, Ji Yeh Choi, Min Song Kim, Gi Hyeong Sin, Sun Ho Hong, Chang Hyun Kim, Seh Hyon Song, Young Wook Choi

**Affiliations:** a College of Pharmacy, Chung-Ang University, Seoul, Republic of Korea; b College of Pharmacy, Keimyung University, Daegu, Republic of Korea; c Department of Psychology, York University, Toronto, Ontario, Canada; d College of Pharmacy, Kyungsung University, Busan, Republic of Korea

**Keywords:** Insulin, oral delivery, self-microemulsifying drug delivery system, D-optimal mixture design, enzyme stability

## Abstract

The lipophilicity of a peptide drug can be considerably increased by hydrophobic ion pairing with amphiphilic counterions for successful incorporation into lipid-based formulations. Herein, to enhance the oral absorption of insulin (INS), a self-microemulsifying drug delivery system (SMEDDS) formulation was developed. Prior to optimization, INS was complexed with sodium *n*-octadecyl sulfate (SOS) to increase the loading into the SMEDDS. The INS–SOS complex was characterized via scanning electron microscopy, Fourier transform infrared spectroscopy, differential scanning calorimetry, and its dissociation behavior. The SMEDDS was optimized using a D-optimal mixture design with three independent variables including Capmul MCM (*X*_1_, 9.31%), Labrasol (*X*_2_, 49.77%), and Tetraglycol (*X*_3_, 40.92%) and three response variables including droplet size (*Y*_1_, 115.2 nm), INS stability (*Y*_2_, 46.75%), and INS leakage (*Y*_3_, 17.67%). The desirability function was 0.766, indicating excellent agreement between the predicted and experimental values. The stability of INS-SOS against gastrointestinal enzymes was noticeably improved in the SMEDDS, and the majority of INS remained in oil droplets during release. Following oral administration in diabetic rats, the optimized SMEDDS resulted in pharmacological availabilities of 3.23% (50 IU/kg) and 2.13% (100 IU/kg). Thus, the optimized SMEDDS is a good candidate for the practical development of oral delivery of peptide drugs such as INS.

## 1. Introduction

Insulin (INS), an intrinsic peptide consisting of 51 amino acids, was first discovered in 1921 by Branting, Best, MacLeod, and Collip (Pillai & Panchagnula, 2001). INS is excreted by the β cells of the pancreas and regulates blood glucose levels by promoting the utilization of glucose in the cells of an organism. INS is prevalently used as a primary anti-diabetic agent. However, its administration route is strictly limited to subcutaneous (SC) or intravenous injection. Since both these injection routes entail pain and inconvenience, patient compliance is an issue (Zhang et al., 2021). Alternatively, diverse approaches have been attempted for INS delivery through various routes. For instance, implantable devices were developed for continuous INS delivery (Bally et al., 2017), and a dry powder was developed for pulmonary INS delivery (Quarta et al., 2020). Although these routes efficiently delivered INS to the systemic circulation, they may require a complicated technique for application and are still inconvenient. In contrast, oral route is preferable because of its ease of administration and safety. Orally delivered INS presents three major advantages: (i) it improves patient compliance; (ii) it undergoes a hepatic bypass before systemic circulation, which can mimic the effects of pancreatic INS by inhibiting hepatic gluconeogenesis and hepatic glucose output (Damgé et al., 2007; Sun et al., 2011); and (iii) it directly transports INS to the liver, thereby preventing peripheral hyperinsulinemia (Sun et al., 2011).

However, oral delivery of INS presents a variety of practical difficulties, including poor absorption from the intestinal lumen because of the high hydrophilicity of INS and the tight junctions in the epithelial lining. In addition, proteases, which exist throughout the entire gastrointestinal (GI) tract, present the greatest obstacle (Verma et al., 2021). To overcome these obstacles, various INS-loaded formulations such as liposomes, nanoparticles, and self-microemulsifying drug delivery systems (SMEDDSs) have been investigated (Zhang et al., 2005; Zhang et al., 2012; Sun et al., 2015). The blood glucose level of diabetic rats was reduced to ∼68% at 2 h after oral administration of INS-loaded nanoparticle composed of poly(lactide-*co*-glycolide) (Sun et al., 2015). Among the formulations, SMEDDSs have been widely used to improve oral bioavailability (BA) of peptide/protein drugs (Hintzen et al., 2014; Menzel et al., 2018). SMEDDSs are an isotropic mixture of oils, surfactants, and cosurfactants (Suram & Veerabrahma, 2022). This mixture spontaneously aggregates and forms nanosized oil-in-water emulsions when diluted with aqueous media such as GI fluids under gentle agitation (Suram & Veerabrahma, 2022). Spontaneous formation of a nanoemulsion incorporates the drugs in the mixture, thereby enhancing their solubilization (Dhaval et al., 2022). Small droplets allow rapid dissolution and enhanced permeation owing to the large surface area (Yao et al., 2008; Zhang et al., 2019).

Although SMEDDSs have already demonstrated considerable potential for developing oral delivery routes for peptide drugs (Cryan, 2005; Zhang et al., 2012), the incorporation of hydrophilic macromolecules into lipophilic matrixes remains a challenge. In particular, since INS has very low lipophilicity, it is difficult to encapsulate it into a lipid matrix (Mahmood & Bernkop-Schnürch, 2019). In addition, even when INS incorporation is successfully achieved, INS leakage from lipid-based formulations (LBFs) is inevitable. Therefore, additional technology is required to increase the lipophilicity of INS. Among the various possible approaches, the hydrophobic ion pairing (HIP) technique has been applied to enhance the hydrophobicity of protein/peptide drugs (Sun et al., 2008; Mahmood & Bernkop-Schnürch, 2019). This technique involves non-covalent bonding in which the charged peptides at a suitable pH interact with oppositely charged surfactants, phospholipids, or other amphiphilic molecules. Taking advantage of increased lipophilicity, HIP complexes of peptides can then be easily incorporated into an SMEDDS. In one study, more than 70% of the HIP complex of INS with dimyristoyl phosphatidylglycerol was protected by SMEDDS from α-chymotrypsin digestion for 3 h (Karamanidou et al., 2015). In addition, a SMEDDS formulation containing a complex composed of INS and soy phosphatidylcholine increased the apical-to-basolateral transportation by approximately threefold in a Caco-2 cell monolayer compared with INS solution (Zhang et al., 2012). By virtue of HIP complexation with sodium oleate, Hintzen et al. (2014) loaded leuprorelin into a SMEDDS formulation, and this SMEDDS showed ∼17-fold increased oral BA compared with leuprorelin solution.

In the past, the optimization of formulations was conducted based on one-factor-at-a-time approaches. However, these empirical approaches are time consuming and may result in inadequate data (Cho et al., 2013). In contrast to traditional trials, application of a design of experiment allows to establish mathematical correlations between input variables and responses by implementing effective statistical approaches (Peres et al., 2017). Among the experimental models, the mixture design takes advantages of evaluating unbiased and precise estimates of the model parameters with a reduced number of experimental runs (Varanda et al., 2017). The basic supposition of mixture design is that, when the process conditions are kept regularly, the response variables are just determined by the proportion of the components in the mixture (Coronado et al., 2014). Furthermore, because the sum of the input variables must be set to 100%, this design is suitable for optimizing the ratio of constituents of the formulations such as SMEDDS (Yeom et al., 2015).

In the present study, in an attempt to improve oral absorption of INS, an HIP complex using INS and sodium *n*-octadecyl sulfate (SOS) was developed for the first time. After the characterization of INS–SOS, the complex was loaded into the SMEDDS formulation. A D-optimal mixture was employed to optimize the proportion of each component using the response variables of droplet size, intestinal enzyme durability, and INS leakage. Furthermore, to evaluate the protective effect of the SMEDDS against GI enzymes, INS stability in the optimized SMEDDS was evaluated after exposure to proteolytic enzymes. Finally, an *in vivo* pharmacodynamic study was performed after oral administration of the optimized SMEDDS to rats.

## 2. Materials and methods

### 2.1. Materials

Human recombinant INS, α-chymotrypsin, bile salts, Brij L4, lipase from porcine pancreas, *n*-octanol, pepsin, Tetraglycol, trypsin, sodium deoxycholate (SDC), sodium docusate (DOC), sodium oleate (OLE), and Tween 20 were obtained from Sigma–Aldrich Chemical Co. (St. Louis, MO, USA). Capmul MCM was purchased from Abitec Co. (Janesville, WI, USA). Capryol 90, Labrasol, Plurol Oleique CC 497, and Transcutol P were supplied by Gattefosse (Saint-Priest, France). Cremophor EL was purchased from BASF (Ludwigshafen, Germany). SOS was obtained from Acros Organics (NJ, USA). All other chemicals and reagents were purchased from commercial sources and were of analytical grade.

### 2.2. Animals

Male Sprague–Dawley rats (200–250 g, 7–9 weeks) were purchased from Orient Bio (Gyeonggi-do, Korea). Prior to the experiments, all rats were allowed to adapt to our legal animal care facility for 1 week. All rats were fasted for 12–18 h overnight prior to either streptozotocin injection or drug administration, with free access to water. All animal trials were performed in compliance with the National Institutes of Health guidelines on the care and use of laboratory animals and were approved by the Institutional Animal Care and Use Committee of Chung-Ang University (Protocol No. A2022022), Seoul, Korea.

### 2.3. High-performance liquid chromatography assay of INS

INS concentration was analyzed using high-performance liquid chromatography (HPLC) as previously reported (Shrestha et al., 2016). Fifty microliters of each sample were injected into an HPLC system (e2695; Waters, Milford, MA, USA) consisting of a pump (W2690/5; Waters), an ultraviolet (UV) detector (W2489; Waters), and a data station (Empower 3; Waters). A C18 column was used (ZORBAX 300SB; 150 × 4.6 mm, 5 μm; Agilent Technologies, Santa Clara, CA, USA), and the mobile system consisted of A [0.1% trifluoroacetic acid (TFA) in distilled water] and B (acetonitrile), with a varied gradient according to the following program: 0 min (80% A), 5 min, (50% A), 7 min (50% A), and 10 min (80% A). Analyses were performed at a flow rate of 1.2 mL/min at 35 °C, and the column eluent was monitored at 214 nm.

### 2.4. Preparation of HIP complexes

According to the previously reported organic solvent-free method (Shahzadi et al., 2020), INS was complexed with various anionic counterions: SDC, OLE, DOC, and SOS. Briefly, the INS solution was prepared in 0.01 M hydrochloride solution, and each counterion was dissolved in distilled water. Thereafter, solutions of each counterion were separately added dropwise to the INS solution while continuously stirring at 800 rpm for 4 h. The resulting complexes were centrifuged at 16,000*g* for 10 min (Smart R17; Hanil Science Industrial, Incheon, Korea). Supernatants were removed, and the obtained complexes were frozen, lyophilized, and stored at −20 °C.

To determine the optimal molar ratio of the counterions to INS, the complexation efficiency (CE) value was calculated using Eq. (1):

(1) $$\mathrm{CE}(\%)=\frac{I_{T}-I_{f}}{I_{T}}\times 100$$

where *I*_T_ and *I*_f_ are the total amount of INS initially added and the amount of INS in the supernatant, respectively.

### 2.5. Determination of log P

The log *P* values of different HIP complexes were determined by the shake-flask method (Zhang et al., 2021). Prior to the experiment, distilled water and *n*-octanol were poured into a separatory funnel, vigorously shaken, and then allowed to settle for 24 h to mutually saturate. Stock solutions of HIP complexes in the saturated *n*-octanol were prepared and mixed with pre-saturated distilled water at a 1:10 (vol/vol) ratio. INS was dissolved in saturated distilled water and then mixed with pre-saturated *n*-octanol. The tube was incubated at 25 °C for 24 h with continuous shaking (50 rpm) and allowed to separate for 1 h, after which it was centrifuged at 16,000*g* for 10 min. Afterward, samples from both phases were carefully analyzed by HPLC. The log *P* value was calculated as follows: log *P* = log (*C_n_*_-octanol_/*C*_aqueous_), where *C_n_*_-octanol_ and *C*_aqueous_ refer to the concentrations of INS in the *n*-octanol and aqueous phases, respectively, at equilibrium.

### 2.6. Characterization of the INS–SOS complex

The solid-state properties of free INS, SOS, a physical mixture (PM) of INS and SOS, and INS-SOS were investigated using scanning electron microscopy (SEM), differential scanning calorimetry (DSC), and Fourier transform infrared spectroscopy (FTIR). The morphological features of each sample were visualized using a scanning electron microscope (Sigma 300; Carl Zeiss Meditec AG, Jena, Germany). The samples were placed on a brass disc using double-sided adhesive carbon tape. A Hitachi ion sputter (E-1030) was employed to coat each sample with platinum under vacuum for 120 s at a current of 4 mA. The samples were scanned at 10 kV. The thermal characteristics of each powder were evaluated using a DSC-Q20 calorimeter (TA Instruments, New Castle, DE, USA). After being sealed in an aluminum pan, each sample (2–3 mg) was subjected to heating in the range of 30–150 °C at a rate of 5 °C/min under a constant nitrogen flow at 25 mL/min. The FTIR spectra were recorded using an FTIR spectrophotometer (Nicolet 6700, Thermo Scientific, USA). Appropriate amounts of the freeze-dried samples were mixed with dry potassium bromide and compressed into discs under 10 kN force at room temperature. The samples were scanned from 4000 to 400 cm^−1^ wavenumber at a resolution of 2 cm^−1^.

### 2.7. Dissociation study of the INS–SOS complex

The dissociation profiles of the INS–SOS complex were evaluated using different concentrations (pH 7.4; NaCl 0 mM, 100 mM, 150 mM, and 200 mM). Briefly, 2 mg of the INS–SOS was weighed in 1.5-mL microtubes, dispersed with 1 mL of phosphate-buffered saline (PBS) solution (pH 7.4, 10 mM), and then incubated at 37 °C. At predetermined time intervals, the samples were centrifuged at 16,000*g* for 5 min. Subsequently, 500 µL of the supernatants was withdrawn and replaced immediately with an equal volume of prewarmed release medium. Finally, the amount of INS within the collected supernatant was analyzed by HPLC, as described earlier.

### 2.8. Far-UV circular dichroism spectroscopy

The INS–SOS complex prepared as described previously was dissolved in PBS (pH 7.4; NaCl 150 mM) at the INS-equivalent concentration of 1 mg/mL. Circular dichroism (CD) measurements were conducted with Chirascan plus Circular Dichroism detector (Appied Photophysics) with 0.05-cm path-length cell, CD spectra were scanned from 195 to 260 nm with 0.2 s integration, 1 nm step resolution, and 1 nm bandwidth at 25 °C. The quantitative analyses were performed using CDNN secondary structure analysis software (version 2.1, authored by Gerald Böhm at the Institute for Biotechnology, Martin Luther University, Halle-Wittenberg, Germany).

### 2.9. Solubility test

The equilibrium solubility of the INS–SOS complex in various vehicles was measured to determine the best oil, surfactant, and cosurfactant for the SMEDDS. Briefly, an excess amount of INS–SOS complex was added to 1 mL of oil, surfactant, and cosurfactant. Each mixture was shaken with a mechanical shaker (CM-1000; EYELA, Tokyo, Japan) at 25 °C for 24 h to reach dynamic equilibrium. The mixtures were centrifuged at 12,000*g* for 10 min to remove excess INS–SOS complex. The supernatant was then appropriately diluted with methanol and filtered through a 0.45-μm polyvinylidene fluoride membrane filter (Whatman International, Kent, UK). Each sample was analyzed via HPLC to determine the concentration of INS.

### 2.10. Construction of a pseudo-ternary phase diagram

The SMEDDS formulations that could self-microemulsify under gentle agitation after dilution were identified using a ternary phase diagram. Based on the results of the solubility study, Capmul MCM, Labrasol, and Tetraglycol were chosen as the oil, surfactant, and cosurfactant, respectively. Each component was allocated to an apex of a triangle. A series of blank SMEDDS formulations was prepared by varying concentrations of three components. For all mixtures, the total proportions of the three components always added up to 100%.

The efficiency of microemulsion formation was assessed by adding 300 μL of each mixture to 3 mL of distilled water and gently stirring it with a magnetic stirrer. The emulsification tendency was judged as ‘‘good’’ when the droplets spread easily in water and formed a fine milky emulsion with the droplet size less than 300 nm and ‘‘bad’’ when there was poor or no emulsion formation with immediate coalescence of oil droplets, especially after stirring was stopped.

### 2.11. Preparation of an HIP-containing SMEDDS

A blank SMEDDS formulation was prepared by mixing oil, surfactant, and cosurfactant. The components were thoroughly mixed at 37 °C to obtain a clear homogenous solution. For the HIP-containing SMEDDS, the lyophilized INS–SOS complex was dissolved in a concentration of 1.4% in the pre-concentrate based on an expected INS payload of 1.0%.

### 2.12. Optimization of the HIP-loaded SMEDDS using a D-optimal mixture design

A D-optimal mixture design was employed to optimize the composition of the HIP-loaded SMEDDS. The oil, surfactant, and cosurfactant were included as independent variables; Capmul MCM (*X*_1_), Labrasol (*X*_2_), and Tetraglycol (*X*_3_) were set within ranges of 5%–20%, 25%–60%, and 35%–70%, respectively. To attain an optimized formulation with a high desirability function, three response variables were adopted: droplet size (*Y*_1_, nm), INS stability against intestinal enzymes (*Y*_2_, %), and INS leakage in pH 1.2 medium (*Y*_3_, %). The design consisted of 17 experimental points to find a model fit, and the mathematical correlations between the inputs and outputs were evaluated using Design-Expert Software version 11 (Stat-Ease Inc., Minneapolis, MN, USA).

### 2.13. Particle size measurement

A dynamic light scattering particle size analyzer (Zetasizer Nano-ZS; Malvern Instruments, UK) was used to determine the droplet size of the nanoemulsion. The definite volume (10 µL) of each SMEDDS was diluted with 10 mL of distilled water followed by gentle vortexing for 1 min. After homogeneous dispersion was obtained, each sample was loaded into a disposable cuvette and placed in a thermostatic chamber at 25 °C. Light scattering was monitored with a 50 mV laser at a 90° angle.

### 2.14. Stability assessment against GI enzymes

Simulated gastric fluid (SGF) was prepared by adding pepsin to pH 1.2 solution to a concentration of 2,000 U/mL. Simulated intestinal fluid (SIF) was prepared by adding trypsin (5,000 BAEE U/mL), chymotrypsin (20 IU/mL), lipase (400 IU/mL), and bile salt (10 mM) to the Tris buffer (50 mM, pH 8.0). Each sample was appropriately diluted to a final INS concentration of 0.2 mg/mL. For the INS and INS–SOS solutions, INS or INS–SOS was dissolved in phosphate buffer (0.05 M, pH 7.0) with 0.05% Tween 20. Then, 250 µL of either SGF or SIF was added to the 250 µL of each formulation for enzymatic degradation. The mixture was transferred to a shaking incubator (SI-900R; JeioTech, Daejeon, Korea) and incubated at 37 °C under shaking (100 rpm). Finally, 500 mL of cooled 0.1% TFA in methanol was added at predetermined time points (0, 5, 15, 30, 60, and 120 min) to quench the enzymatic reaction. For intestinal enzyme study, the reaction was continued for 240 min. Finally, the amount of INS remaining in the obtained samples was quantified by HPLC, as described earlier.

### 2.15. In vitro drug release

An *in vitro* drug release study was performed using a dialysis bag diffusion method. Briefly, samples of each preparation containing 1 mg of INS equivalent were placed in a dialysis bag (300 kDa MWCO; Spectrum Laboratories, Rancho Dominguez, CA, USA). Then, firmly clipped dialysis bags were soaked in 30 mL of either pH 1.2 solution with Labrasol (1%, wt/wt) or pH 6.8 solution while stirring at 100 rpm, after which they were incubated for 8 h. For the measurement of the amount of released INS, 1 mL of the release medium was withdrawn at predetermined time points (0.5, 1, 2, 3, 4, 6, and 8 h), and the dialysate volume was replenished with 1 mL of fresh release medium. The concentration of INS in the aliquots was analyzed by HPLC, as described earlier.

### 2.16. Pharmacodynamic study in diabetic rats

To establish animal models of type 1 diabetes mellitus, male Sprague–Dawley rats were injected intraperitoneally with freshly prepared single doses of streptozotocin (60 mg/kg) dissolved in citrate buffer (0.1 M, pH 4.5). The successful induction of diabetes was corroborated by an increase in blood glucose levels after five days. Only the rats with fasting blood glucose levels higher than 250 mg/dL were considered diabetic.

To evaluate and compare the pharmacodynamic efficacy of the different formulations, the diabetic Sprague–Dawley rats were randomly divided into five groups (*n* = 5–7 rats per group): Group 1 received INS solution, Group 2 received INS–SOS solution, Group 3 received the optimized SMEDDS at a dose of 50 IU/kg (SMEDDS-50IU), Group 4 received the optimized SMEDDS at a dose of 100 IU/kg (SMEDDS-100IU), and Group 5 received INS (SC). All rats were fasted for 12 h before the experiment, with free access to water. Each INS test sample was directly administrated via oral gavage, except in Group 5. The INS–SOS solution was prepared as described in Section 2.12. Next, to compare the hypoglycemic effect with the standard, the INS solution was injected subcutaneously to the rats in Group 5 at a dose of 5 IU/kg. Subsequently, blood samples were collected from the tail vein at different time intervals (0, 0.5, 1, 1.5, 2, 3, 4, 5, 6, and 8 h) and tested using a glucometer (GreenDoctor, GREENCROSS MEDIS Co., Chungnam, Korea). Based on the blood glucose level at 0 h (initial), each blood glucose level was converted to the relative level (% of initial). A value of 100% of the relative level was chosen as a reference point (upper limit) for the determination of the area above the blood glucose (% of initial)–time curve (AAC) values for decreased blood glucose levels. After calculating AAC values using the trapezoidal rule, the pharmacological availability (PA) relative to the SC administration of INS was calculated using Eq. (2) as follows:

(2) $$\mathrm{PA}[\%]=\frac{{\mathrm{AAC}}_{\mathrm{oral}}}{{\mathrm{AAC}}_{\mathrm{sc}}}\times \frac{{\mathrm{Dose}}_{\mathrm{sc}}}{{\mathrm{Dose}}_{\mathrm{oral}}}\times 100$$

where AAC_oral_, AAC_SC_, Dose_oral_, and Dose_SC_ refer to the AAC of oral administration, AAC of SC administration, dose of oral administration, and dose of SC administration, respectively.

### 2.17. Statistical analysis

All data are presented as the mean ± standard deviation (SD). Data analysis was performed with one-way independent groups analysis of variance (ANOVA) followed by Tukey’s test for post hoc comparison. For all analyses, differences were considered significant when the *p* values were less than .05, unless otherwise indicated.

## 3. Results and discussion

### 3.1. Screening of anionic counterions

**Figure 1.**
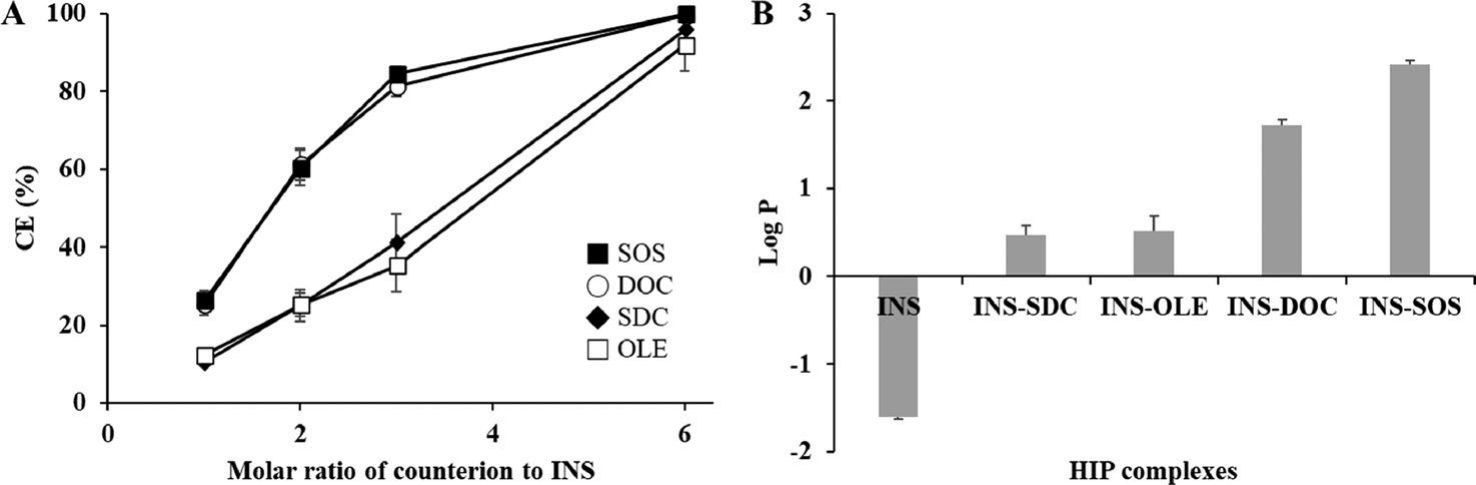
Physical characterization of HIP complexes. A: Complexation efficiency of INS with different counterions at the indicated counterion to INS molar ratio, (B) partition coefficient of the HIP complex of INS. Values are presented as mean ± standard deviation (*n* = 3). CE, complexation efficiency; INS, insulin; SOS, sodium *n*-octadecyl sulfate; DOC, sodium docusate; SDC, sodium deoxycholate; OLE, sodium oleate; HIP, hydrophobic ion pairing.

INS was complexed with various amphiphilic molecules. The CE values of INS with each counterion are depicted in Figure 1(A). With an increasing concentration of counterions, the CE values increased until a maximum was reached. All counterions showed a maximum CE value (>90%) when the molar ratio of INS to counterion was 1:6, which corresponds with the number of positively charged groups of INS (Sun et al., 2011). The maximal CE values of SDC and OLE were found to be lower than those of other counterions, with values of 95.81% and 91.79%, respectively. This result indicated that carboxylic acid derivatives were not suitable for complexation with INS. Carboxylic acid derivatives are easily protonated in acidic conditions because of their relatively high p*K*_a_ values (>5) (Noh et al., 2022). The INS solution was prepared in 0.01 M HCl solution; thus, these counterions were vulnerable to being protonated and thus losing their ability to interact with INS (Ristroph & Prud’homme, 2019). In general, carboxylic acid derivatives cannot be recommended for HIP complexes because HIP complexes must be prepared in an acidic environment (Ristroph & Prud’homme, 2019). In contrast, DOC and SOS showed sufficiently high CE values (>97%), confirming the successful formation of an HIP complex.

To evaluate the increase in lipophilicity as a result of HIP complexation, the log *P* values were determined. Free INS had a low log *P* value of –1.61, as illustrated in Figure 1(B). Based on the CE values, the molar ratio of INS to counterions was fixed at 1:6. Following HIP complexation, the lipophilicity of INS increased considerably, owing to the attachment of the lipophilic molecules. The lipophilicity of HIP complexes paired with SDC and OLE was found to be relatively low. This might be due to the acidic conditions in which the HIP complexes were prepared; because the negative charges of carboxylic acid derivatives were insufficient in this condition, unstable HIP complexes could be formed. In contrast, DOC and SOS produced HIP complexes with higher log *P* values. As these counterions have sufficiently low p*K*_a_ values, they could become more tightly bound to INS, resulting in more lipophilic complexes (Shahzadi et al., 2020). Of all the complexes, INS–SOS complex showed the highest lipophilicity. In general, the more hydrophobic counterions that are attached, the more lipophilic complexes are formed (Nazir et al., 2019). Due to the high acidity and high log *P* value of SOS, the resulting complex exhibited a high log *P* value. Thus, the development of the SMEDDS formulation was fulfilled using the INS–SOS complex.

### 3.2. *Characterization of the INS*–*SOS complex*

**Figure 2.**
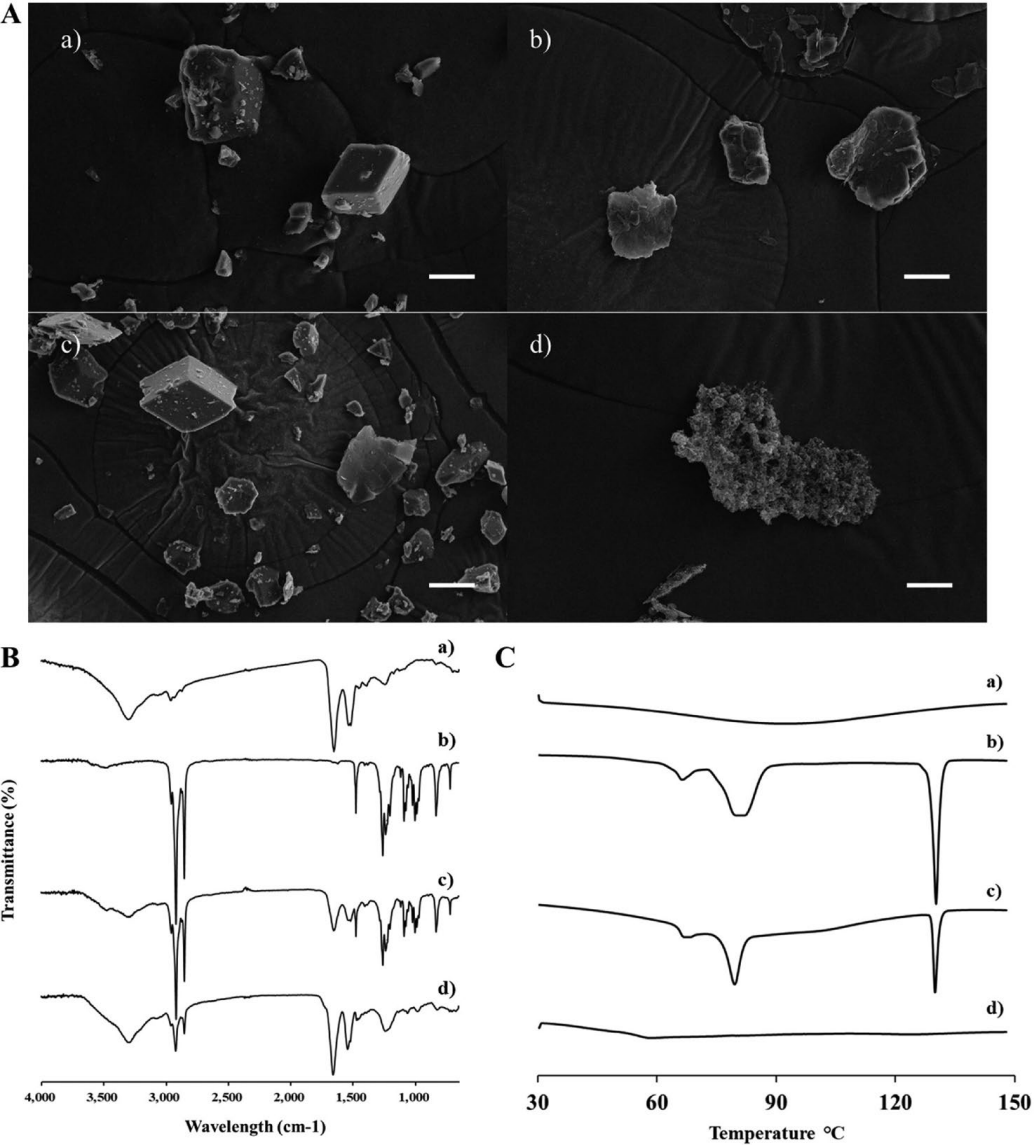
Characterization of INS–SOS complex. A: Scanning electron microscopy; (B) Fourier transform infrared spectroscopy; (C) differential scanning calorimetry thermograms. (a) INS; (b) SOS; (c) physical mixture of INS and SOS; (d) INS–SOS. Scale bar = 5 μm. INS, insulin; SOS, sodium *n*-octadecyl sulfate.

The formation of the INS–SOS complex was confirmed using SEM, FTIR, and DSC. The morphological aspects of each sample were visualized using SEM (Figure 2(A)). Free INS was shown to be an irregular lump crystal with a smooth surface, as previously reported (Zhao et al., 2010). SOS appeared to have an indefinite shape and size. In the PM, the typical morphologies of both free INS and SOS were present. No distinct crystallinity of INS was observed in INS–SOS, which instead presented as a lumped mass with a rough surface, thus indicating that the complex had been successfully formed. As shown in Figure 2(B), the FTIR spectrum of INS was found to have four characteristic absorption peaks: 3300 cm^−1^ (amide A), 1645 cm^−1^ (amide I), 1515 cm^−1^ (amide II), and 1240 cm^−1^ (amide III). The spectral analysis of SOS showed the presence of distinct peaks at 1256 cm^−1^, 1233 cm^−1^, and 1200 cm^−1^ attributed to –SO_2_ asymmetric stretching; peaks at 1085 cm^−1^ and 1071 cm^−1^ attributed to –SO_2_ symmetric stretching; and asymmetric and symmetric stretching corresponding to the CH_2_ of the acyl chains at 2916 cm^−1^ and 2850 cm^−1^, respectively. The PM of INS and SOS preserved the distinct vibrational bands of both INS and SOS. However, these absorption bands disappeared in INS–SOS. Moreover, the amide peaks of INS remained, suggesting that the secondary structures of INS were retained. Furthermore, DSC analysis was conducted to evaluate the crystalline property of INS–SOS. DSC thermograms are shown in Figure 2(C). There were no specific endothermic peaks for INS, as previously reported (Bashyal et al., 2021). In contrast, SOS had a sharp endothermic peak at ∼130 °C. The PM yielded an endothermic peak corresponding to that of SOS; however, INS–SOS exhibited no endothermic peak at the melting point of SOS. These results implied successful complexation between INS and SOS.

### 3.3. *Effect of NaCl on dissociation of the INS*–*SOS complex*

**Figure 3.**
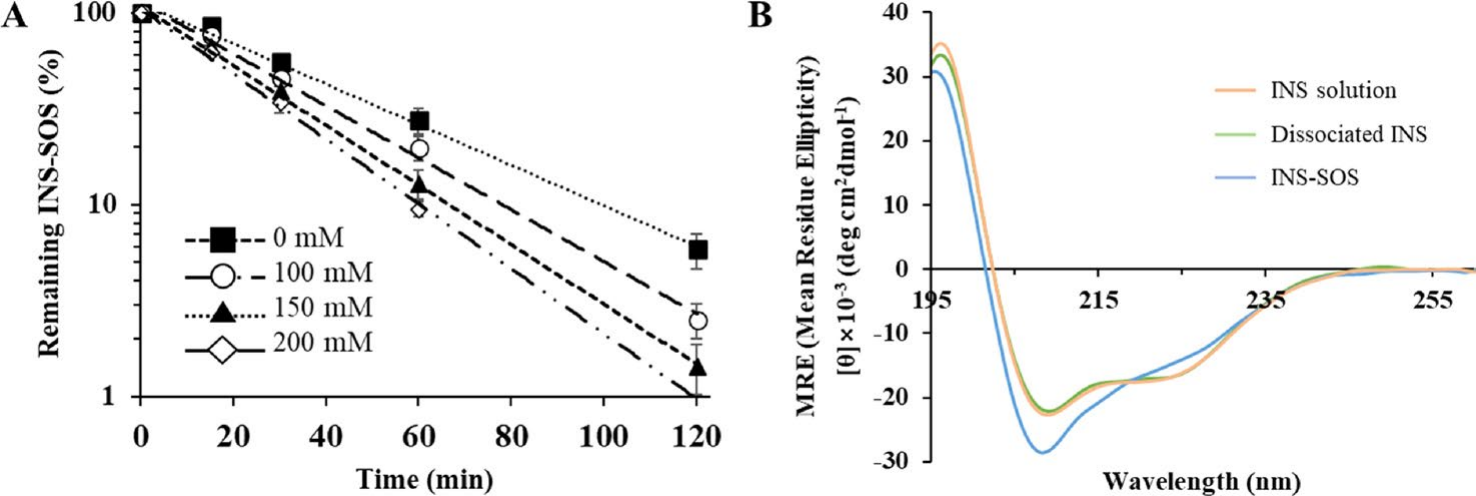
Dissociation behavior of the INS–SOS complex. A: Semi-log plots of the remaining INS–SOS complex in different concentrations of NaCl; (B) far-ultraviolet circular dichroism spectra of the INS solution, the dissociated INS, and INS–SOS. Values are presented as the mean ± standard deviation (*n* = 3). INS, insulin; SOS, sodium *n*-octadecyl sulfate.

The dissociation behavior of the HIP complex was assessed at different NaCl concentrations. As shown in Figure 3(A), the semi-log plot of the remaining INS versus time was a straight line with good linearity (*R*^2^ > 0.9), indicating the first-order degradation of INS according to the following equation [Eq. (3)]:

(3) $$\frac{C}{C_{0}}=e^{-kt}$$

where *C*/*C*_0_ is the fraction of remaining INS–SOS at the predetermined time point *t*, and *k* is the rate constant (min^−1^). The regression function of Microsoft Excel was used to obtain the *k* values, which were 0.0244 (0 mM), 0.0313 (100 mM), 0.0360 (150 mM), and 0.0392 (200 mM). Even when NaCl was not included, INS was dissociated from the complex, with a half-life of under 30 min (Supplementary Table S1). As the concentration of NaCl increased, the dissociation process was promoted, resulting in reduced half-lives. This might be due to the ion exchange between the HIP complex and electrolytes. In general, salt concentration promotes the dissociation of HIP complexes, leading to the leakage of encapsulated peptides from LBFs (Chamieh et al., 2019). Likewise, NaCl salt destabilized the INS–SOS complex. In the isotonic condition (150 mM), the dissociation process was noticeably promoted, with a half-life of ∼13 min. These results indicated that INS molecules could be rapidly released from the INS–SOS complex and to exert the pharmacological effect of INS after attaining systemic circulation.

**Table 1.** Secondary structural compositions of the INS solution, dissociated INS, and INS-SOS.

	α helix	Antiparallel	Parallel	β turn	Others
INS	38.0%	6.9%	7.7%	16.0%	29.8%
Dissociated INS	36.7%	7.4%	7.9%	16.2%	30.6%
INS-SOS	39.4%	7.3%	6.9%	16.1%	25.6%

The quantitative analysis was performed using CDNN secondary structure analysis software. INS, insulin; SOS, sodium *n*-octadecyl sulfate.

### 3.4. Structure integrity of INS in the complex

Secondary structures, which are the backbone of a protein/peptide, are a prominent feature required for the stability of peptide drugs. Changes in the protein structure could raise concerns of decreased pharmacological activity and toxicological issues (Bashyal et al., 2021). Thus, to confirm the secondary structure of INS dissociated from the INS–SOS complex, the far-UV CD spectroscopies were determined. The CD spectrum of the INS solution showed two strong bands at about 209 nm and 223 nm, suggesting the existence of distinct α-helical structures and a β-structure, respectively (Amaral et al., 2020). As shown in Figure 3(B), the far-UV spectrum of the INS–SOS complex was slightly different from that of the INS solution, whereas the spectrum of INS dissociated from INS–SOS was similar to that of the INS solution. This suggested that the secondary structure of INS was slightly altered as a result of SOS binding to INS; however, the secondary structure of INS was recovered after it was detached from SOS. This result corresponded closely to the findings of a previous report, in which the secondary structure of INS dissociated from the HIP complex paired with SDC was unchanged compared with that in the INS solution (Dai & Dong, 2007). Furthermore, the constituents of the secondary structures of the INS–SOS complex and the INS released from INS–SOS complex were compared quantitatively with those of the INS solution (Table 1). The quantitative analysis demonstrated that the INS–SOS complex was composed of 39.4% α-helix, 14.2% β-sheet, 16.1% turn, and 25.6% other secondary structures; the dissociated INS was composed of 36.7% α-helix, 15.3% β-sheet, 16.2% turn, and 30.6% other secondary structures, which is consistent with the 38.0% α-helix, 14.6% β-sheet, 16.0% turn, and 29.8% other secondary structures present in the INS solution. From the CD spectra, the formation of the INS–SOS complex was stable, signifying that the secondary structures were not regained after complexation. In summary, the INS–SOS complex retained the secondary structures of INS; therefore, it is expected to be rapidly dissociated and display anti-diabetic effects after being absorbed.

### 3.5. Screening of lipid vehicles

To successfully develop a SMEDDS formulation containing hydrophobic molecules, the selection of lipid vehicles with high drug-solubilizing capacities is highly recommended (Liao et al., 2019). The oil assumes a key role in solubilizing hydrophobic materials, and both the surfactant and cosurfactant form a stable nanodispersion. The solubility of INS–SOS in various lipid vehicles is listed in Table 2. Compared with other oils, Capmul MCM showed the highest INS–SOS solubility and was therefore selected as the oil. Labrasol and Tetraglycol were selected as the surfactant and cosurfactant, respectively, because of their greater INS–SOS solubility than that of other excipients. Thus, the SMEDDS formulation was further developed using Capmul MCM, Labrasol, and Tetraglycol as the oil, surfactant, and cosurfactant, respectively.

**Table 2.** Solubility of INS-SOS in various lipid vehicles.

Vehicle	Solubility (mg/mL)
Oil	
Capryol 90	6.41 ± 0.17
Capmul MCM	14.40 ± 0.54
Plurol Oleique CC 497	10.56 ± 0.33
Surfactant	
Tween 20	0.75 ± 0.06
Labrasol	1.09 ± 0.05
Cremophor EL	0.46 ± 0.02
Cosurfactant	
Tetraglycol	45.11 ± 1.24
Transcutol P	36.54 ± 2.87
Brij L4	7.74 ± 0.58

Values are presented as the mean ± standard deviation (*n* = 3). INS, insulin; SOS, sodium *n*-octadecyl sulfate.

### 3.6. Pseudo-ternary phase diagram

**Figure 4.**
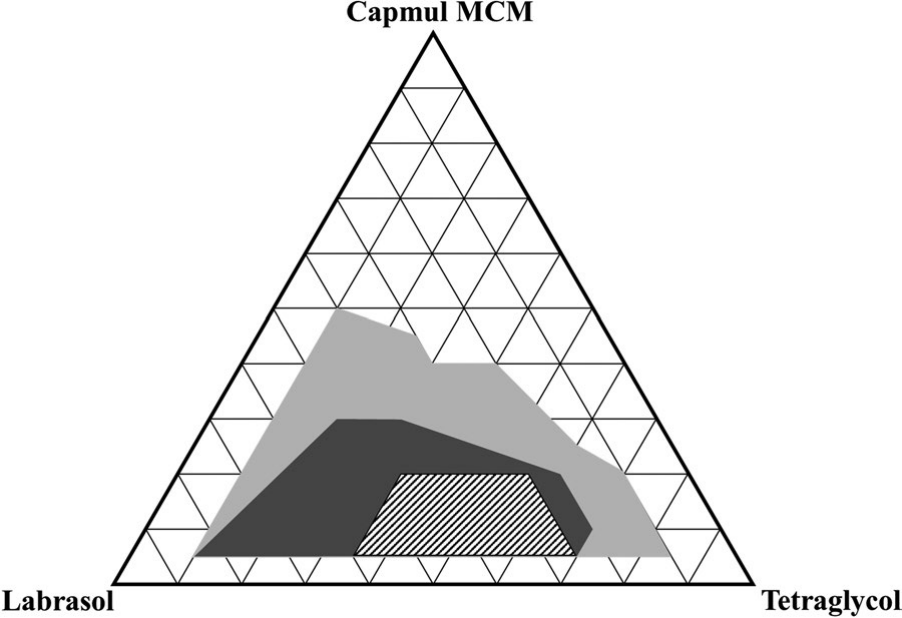
Pseudo-ternary phase diagram of Capmul MCM (oil), Labrasol (surfactant), and Tetraglycol (cosurfactant). Light gray, dark gray, and dashed areas indicate regions for the self-emulsifying drug delivery system, self-microemulsifying drug delivery system, and experimental domain, respectively.

When developing a SMEDDS formulation for water-insoluble molecules such as the INS–SOS complex, it is important to determine the optimal ratio between lipid components (Patel & Sawant, 2019). A pseudo-ternary phase diagram was constructed using Capmul MCM, Labrasol, and Tetraglycol in drug-free conditions. As shown in Figure 4, the SMEDDS regions (dark gray area) were developed using a volume ratio of 5%–25% oil, 20%–85% surfactant, and 10%–80% cosurfactant, which yielded homogeneous and nanosized droplets (<300 nm) with a transparent or bluish white appearance. Although all the preparations in the gray area had self-emulsifying capacity within 1 min, the SMEDDS region was selected for further study because it demonstrated the highest self-emulsifying capacity. It was also found that emulsions were extremely unstable when the proportion of oil exceeded 50%.

It has been reported that the drug loading may also affect self-microemulsifying performance (Lee et al., 2014). The INS–SOS complex was added to 1% [wt/wt] in INS-equivalent for the selected SMEDDS system to ensure the efficient loading of the complex. Some formulations with a low surfactant or cosurfactant ratio resulted in aggregation or precipitation of INS–SOS due to low solubilizing capacity (data not shown). Thus, these marginal regions with too low or too high a surfactant or cosurfactant ratio were excluded, and the experimental domain was finally determined as the dashed area shown in Figure 4.

### 3.7. Statistical analysis using a D-optimal mixture design

**Figure 5.**
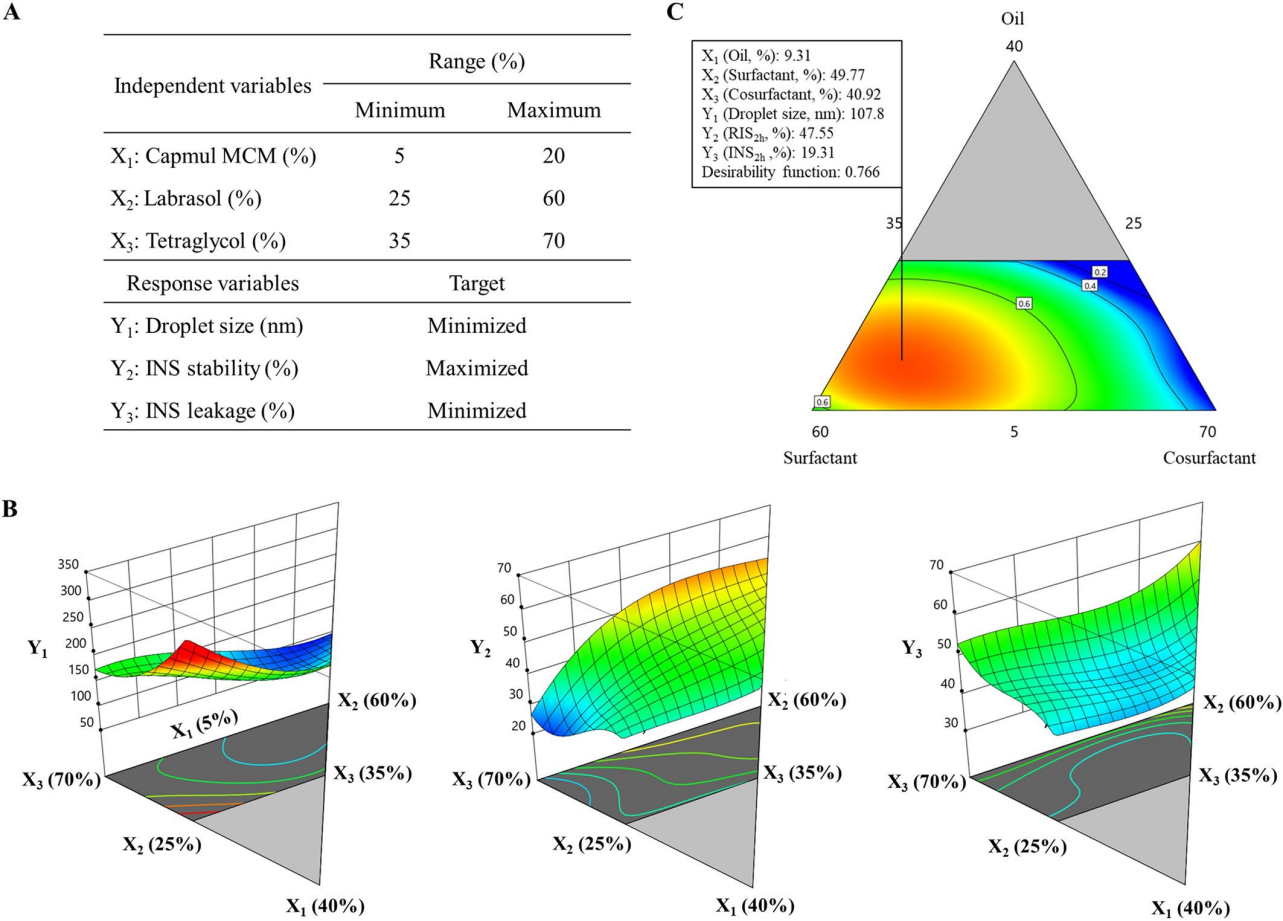
Experimental design and mathematically analyzed plots. A: Independent and response variables used in the D-optimal mixture design. (B) Three-dimensional response surface plots of each response variable. (C) Overlay plot of the optimized self-microemulsifying drug delivery system formulation. Values in contour lines represent the desirability; *X*_1_: Capmul MCM, *X*_2_: Labrasol, *X*_3_: Tetraglycol, *Y*_1_: droplet size, *Y*_2_: INS stability, *Y*_3_: INS leakage. INS, insulin.

A D-optimal mixture design was adopted to optimize the INS–SOS-loaded SMEDDS formulation. In this design, the variance is associated with the evaluation of coefficients in a model, and this produces the best possible subset by considering the criteria for maximizing the information matrix determinants (Mura et al., 2005). Moreover, the D-optimal mixture design considers the total SMEDDS system as 100%, unlike other designs such as Box–Behnken and factorial designs (Yeom et al., 2015). Based on the results from the pseudo-ternary phase diagram, Capmul MCM (*X*_1_; 5%–20%), Labrasol (*X*_2_; 25%–60%), and Tetraglycol (*X*_3_; 35%–70%) were selected as the independent variables (Figure 5(A)), while droplet size (nm; *Y*_1_), INS stability (%; *Y*_2_), and INS leakage (%; *Y*_3_) were selected as the response variables. Droplet size reflects how well the microemulsion formed, INS stability shows the resistance to enzymatic degradation in the small intestine, and INS leakage describes the drug-holding capacity of microemulsions after oral administration. As listed in Supplementary Table S2, 17 trials were performed in accordance with the suggested experimental runs. *Y*_1_ ranged from 95.8 to 244.4 nm, *Y*_2_ from 25.15% to 57.12%, and *Y*_3_ from 15.58% to 39.29%. Thereafter, the experimental results were input into the following four models: linear, quadratic, special cubic, and cubic, and their statistical parameters were calculated (Table 3). The SD signifies the amount of variation or dispersion of a set of values and shows how well the experimental values suit the current model. The predicted residual error sum of squares (PRESS) is also used to determine how well a given model fits the data. The model with the lowest SD and PRESS values is deemed the most desirable (Son et al., 2018). A higher *R*^2^ implies higher prediction accuracy of the fitted model, and the model with the highest *R*^2^ value is deemed the most desirable. Furthermore, because a significant lack of fit measure is indicative of a large deviation between the results and the fitted model, a good model should denote a statistically nonsignificant lack of fit measurement. Given this, all of the statistical parameters indicated that the cubic model was most suitable for further optimization.

**Table 3.** Summary of the results of the statistical analysis and model equations for the measured responses.

Model	SD	*R* ^2^	*R*^2^ (adj)	*p* value	Lack of fit *p* value	PRESS	Remark
Droplet size (nm)							
Linear	17.53	0.8807	0.8755	<.0001	<.0001	16,920.15	–
Quadratic	9.25	0.9689	0.9653	<.0001	.0003	5,123.73	–
Special cubic	6.95	0.9829	0.9804	<.0001	.0086	2,928.82	–
Cubic	3.54	0.9959	0.9949	<.0001	.9668	8,13.19	Suggested
INS stability (%)							
Linear	5.16	0.7581	0.7476	<.0001	<.0001	1,523.40	–
Quadratic	2.70	0.9383	0.9312	<.0001	.0119	433.10	–
Special cubic	2.29	0.9567	0.9505	.0001	.0659	312.15	–
Cubic	1.77	0.9759	0.9703	<.0001	.4758	207.19	Suggested
INS leakage (%)							
Linear	3.84	0.7333	0.7217	<.0001	.0107	831.44	–
Quadratic	2.30	0.9103	0.8999	<.0001	.6858	324.25	–
Special cubic	2.23	0.9176	0.9058	.0616	.7628	312.77	–
Cubic	2.02	0.9372	0.9227	.0131	.9464	262.17	Suggested

INS, insulin; SD, standard deviation; PRESS, predicted residual error sum of squares.

### 3.8. Effect of independent variables on the responses in experimental design

Normal plots of residuals and externally studentized residuals were utilized to assess the adequacy of the cubic model. As shown in Supplementary Figure S1(A), a straight line was found in the normal probability plots for residuals since the underlying error allotment was normally distributed, which indicates that the normality assumptions were satisfied. The residuals were focused on the middle of the straight line, suggesting that the results of the experimental runs were distributed normally. Supplementary Figure S1(B) shows the estimations of the externally studentized residuals, and outlier data were not obtained for this range. The structureless and randomly distributed pattern confirmed that the test did not rely on time or constant variance (Ashar et al., 2021).

Table 4 shows the results of ANOVA for each response. The *F*-test for the linear effect parameter showed a significant difference, indicating that at least one independent variable resulted in a significant effect on the responses (Son et al., 2018). According to the suggested parameters, the polynomial regression equations were calculated as follows:

(4) $$Y_{1}=1043.07X_{1}+99.83X_{2}+170.87X_{3}-1421.82X_{1}X_{2}-985.32X_{1}X_{3}-4.47X_{2}X_{3}-55.82X_{1}X_{2}X_{3}-668.06X_{1}X_{2}(X_{1}-X_{2})-112.71X_{1}X_{3}(X_{1}-X_{3})-132.82X_{2}X_{3}(X_{2}-X_{3})$$

(5) $$Y_{2}=-288.45X_{1}+53.91X_{2}+26.42X_{3}+562.25X_{1}X_{2}+635.71X_{1}X_{3}+53.39X_{2}X_{3}-825.09X_{1}X_{2}X_{3}+280.75X_{1}X_{2}(X_{1}-X_{2})+403.79X_{1}X_{3}(X_{1}-X_{3})-20.60X_{2}X_{3}(X_{2}-X_{3})$$

(6) $$Y_{3}=-220.42X_{1}+37.63X_{2}+29.73X_{3}+421.57X_{1}X_{2}+424.91X_{1}X_{3}-21.49X_{2}X_{3}-607.38X_{1}X_{2}X_{3}+345.76X_{1}X_{2}(X_{1}-X_{2})+261.36X_{1}X_{3}(X_{1}-X_{3})-21.77X_{2}X_{3}(X_{2}-X_{3})$$

**Table 4.** Analysis of variance for the quadratic model of the experimental responses.

		*Y*_1_ (Droplet size)			*Y*_2_ (INS stability)			*Y*_3_ (INS leakage)		
Source	DF	SS	*F* value	*p* value	SS	*F* value	*p* value	SS	*F* value	*p* value
Model	9	0.01	1044.02	<.0001	4948.13	175.16	<.0001	2382.11	64.70	<.0001
Linear mixture	2	0.01	4154.77	<.0001	3844.05	612.36	<.0001	1863.71	227.80	<.0001
*X*_1_ *X*_2_	1	125.73	10.02	.0030	19.66	6.26	.0166	11.05	2.70	.1083
*X*_1_ *X*_3_	1	64.31	5.12	.0292	26.77	8.53	.0058	11.96	2.92	.0952
*X*_2_ *X*_3_	1	5.06	0.40	.5292	722.38	230.15	<.0001	117.07	28.62	<.0001
*X*_1_ *X*_2_ *X*_3_	1	0.1678	0.01	.9085	36.66	11.68	.0015	19.87	4.86	.0335
*X*_1_*X*_2_ (*X*_1_ – *X*_2_)	1	77.31	6.16	.0175	13.65	4.35	.0436	20.71	5.06	.0302
*X*_1_*X*_3_ (*X*_1_ – *X*_3_)	1	2.55	0.20	.6548	32.70	10.42	.0025	13.70	3.35	.0749
*X*_2_*X*_3_ (*X*_2_ – *X*_3_)	1	760.77	60.61	<.0001	18.30	5.38	.0205	20.44	5.00	.0312
Residual	39	489.50	–	–	122.41	–	–	159.54	–	–
Lack of fit	24	200.73	0.4344	.9668	76.65	1.05	.4758	69.45	0.4819	.9464
Pure error	15	288.77	–	–	45.76	–	–	90.09	–	–
Total	50	0.01	–	–	5556.42	–	–	2963.55	–	–

*X*_1_: Capmul MCM, *X*_2_: Labrasol, *X*_3_: Tetraglycol. INS, insulin; DF, degree of freedom; SS, sum of square.

Each linear or polynomial regression coefficient represents the relationship between the independent variables and responses. The signs in front of each regression coefficient indicate a positive or negative relationship, and a larger absolute value of a regression coefficient indicates a stronger impact on the responses (Tang et al., 2021).

Among all of the linear effects, oil was the major factor for all responses (Figure 5(B)). For successful encapsulation of hydrophobic molecules into the SMEDDS, the oil phase assumed a crucial role. As the lipophilicity of INS increased by forming the INS–SOS complex, the SMEDDS should provide sufficient lipidic space to the complex. For *Y*_1_, three independent variables showed a positive effect, indicating that all the SMEDDS constituents were involved in the formation of microemulsion. An emulsion is a mixture of two different liquids that are immiscible, and in the SMEDDS, the oil phases were separated by surfactants. Thus, the more of the oil component that was included, the larger the droplets that were formed. The droplet size of previously developed SMEDDS formulations containing Capmul MCM as an oil phase increased when the oil proportion increased (Park et al., 2021; Goo et al., 2022). The surfactant and cosurfactant stabilize the interfacial membrane of the microemulsion, leading to the formation of smaller microemulsions. Therefore, the effects of both *X*_2_ and *X*_3_ on *Y*_1_ were not great as that of *X*_1_.

*X*_1_ negatively impacted *Y*_2_, indicating that the presence of the oil phase was disadvantageous for enzymatic degradation. SIF contains lipase and bile salts, which act as major barriers to oral delivery of LBFs; therefore, the oil in the SMEDDS is susceptible to degradation in SIF. In general, this digestion process affected the oil phase more significantly than the surfactant and cosurfactant phases (Yin et al., 2009). As the oil phase collapsed, the entrapped INS–SOS complex was exposed to intestinal enzymes such as trypsin and α-chymotrypsin. In contrast, the surfactant had a positive effect on *Y*_2_. Surfactants have high hydrophilicity and can thus stabilize the interface of microemulsions and block these enzymes from approaching. Several reports have demonstrated the great resistance of Labrasol to the pancreatic lipase of human and porcine origin (Fernandez et al., 2007; Nazir et al., 2019). Labrasol, a lipid-based self-emulsifying excipient, is mainly composed of polyethylene glycol (PEG) esters and glycerides with medium acyl chains. In nanoparticles containing PEG emulsifiers, the amount of digested lipid decreased as the PEG concentration increased (Ban et al., 2018). Likewise, a SMEDDS formulation containing Labrasol was found to show great resistance to pancreatic lipase. Tetraglycol also had a positive effect on *Y*_2_. By stabilizing the interface of oil droplets, Tetraglycol could protect the microemulsions from GI enzymes. Thus, *X*_3_ had a positive effect on *Y*_2_.

In the case of *Y*_3_, INS leakage decreased as *X*_1_ increased, indicating that the INS–SOS complex successfully incorporated into the oil phase once microemulsion was generated. Capmul MCM could hold onto the HIP complex and prevent it from leaking due to its solubilizing capacity. The INS–SOS complex was also highly solubilized in Tetraglycol. However, *X*_3_ was positively correlated with *Y*_3_. In general, HIP complexes of protein drugs showed high solubility in these cosurfactants/co-solvents such as Tetraglycol (Griesser et al., 2017). However, as these glycols tend to be rapidly released from the microemulsion, INS leakage occurred during the experiment (Wibel et al., 2020). Hence, a positive effect was found between *X*_3_ and *Y*_3_. In addition, *X*_2_ showed a positive effect on *Y*_3_, which might be due to the solubilizing effect of the surfactant. Since surfactants are capable of solubilizing hydrophobic molecules, the released amount of the drug increases when surfactants are added to the system. However, the magnitude of contribution was relatively small.

### 3.9. *Optimization of the INS*–*SOS-loaded SMEDDS using a desirability function*

The three responses were optimized using appropriate targets. Smaller nanodispersions are known to be absorbed more easily in the GI tract than larger dispersions; thus, the droplet size (*Y*_1_) was set as minimized. To obtain a pharmacological effect from INS, microemulsions must protect the INS–SOS complex from enzymatic attack. Microemulsions should also prevent INS release, thereby achieving enhanced intestinal absorption. At these points, *Y*_2_ was set as maximized, and INS leakage (*Y*_3_) was set as minimized to accomplish successful oral INS delivery.

Based on a desirability function, three independent variables were optimized to satisfy the set targets. As shown in Figure 5(C), *X*_1_, *X*_2_, and *X*_3_ were optimized as 9.31%, 49.77%, and 40.92%, respectively, with a corresponding desirability function value of 0.766. The predicted and observed values of each response for the optimized SMEDDS are shown in Table 5. The prediction errors were calculated to evaluate the reliability and accuracy. The prediction errors of each response were lower than 10%, confirming that the D-optimal mixture design with the cubic model accurately optimized the INS–SOS-loaded SMEDDS. Thus, this optimized product was subjected to further studies.

**Table 5.** Predicted and experimental values for the optimized SMEDDS formulation.

	*Y*_1_ (Droplet size)	*Y*_2_ (INS stability)	*Y*_3_ (INS leakage)
Predicted value	107.8	47.56	19.31
Experimental value	115.2 ± 3.7	46.75 ± 3.04	17.67 ± 2.22
Prediction error (%)	6.86	–1.73	–9.28

Values are presented as the mean ± standard deviation (*n* = 3). SMEDDS, self-microemulsifying drug delivery system.

### 3.10. Protection of INS from enzymatic attack by GI enzymes

**Figure 6.**
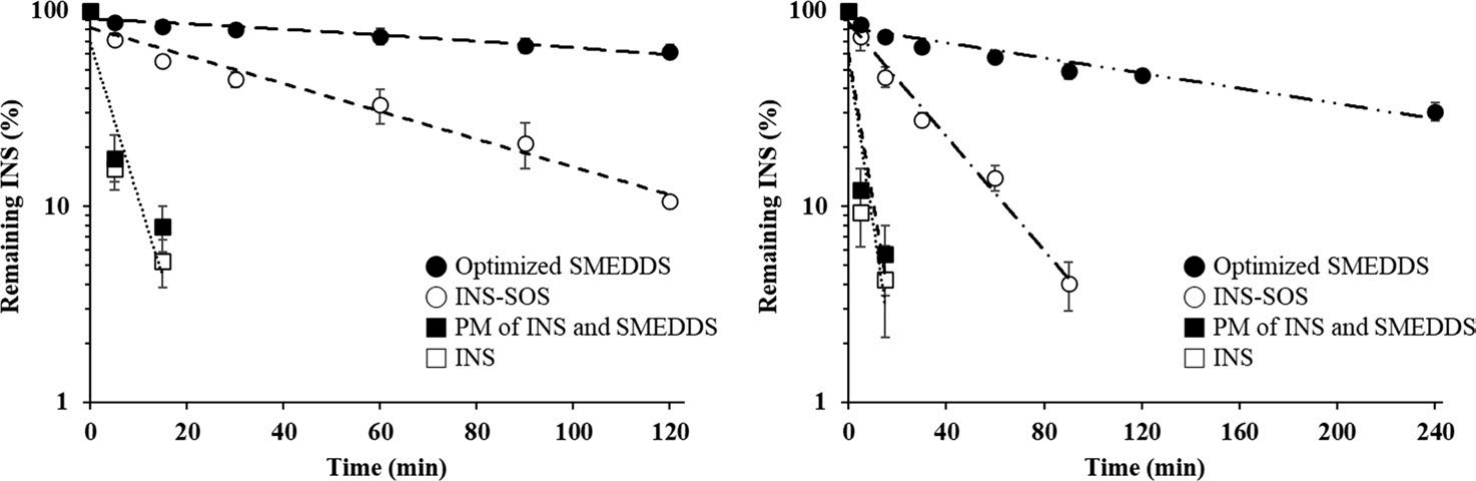
Semi-log plots of remaining INS in different formulations in simulated gastric fluid (left) and simulated intestinal fluid (right). Values are presented as the mean ± standard deviation (*n* = 3). INS, insulin; SOS, sodium *n*-octadecyl sulfate; PM, physical mixture; SMEDDS, self-microemulsifying drug delivery system.

The high enzymatic activity of the GI tract represents a major obstacle to the delivery of therapeutic peptides (Haddadzadegan et al., 2022). The absorption of peptide drugs is strictly limited by the presence and action of numerous peptidases that provoke proteolysis in both the stomach and intestines. Thus, to obtain a pharmacological effect from peptide drugs, lipid-based carriers should be employed to protect peptides from GI enzymes. The INS stability of various formulations in the presence of GI enzymes was evaluated. Figure 6 represents the semi-log plot of the remaining INS versus time. As expected, the INS solution was rapidly degraded by the proteolytic enzymes. The calculated *k* values of the INS solution were 0.1854 min^−1^ for SGF and 0.1980 min^−1^ for SIF, with corresponding half-lives of 3.74 and 3.50 min, respectively (Supplementary Table S3). This indicated that free INS was extremely unstable in the GI tract. The half-lives of the INS–SOS complex were slightly decreased compared with those of the INS solution, suggesting that their resistance to GI enzymes had been improved due to HIP complexation. Furthermore, the *k* value of INS–SOS in SGF was lower than that in SIF. This might be due to the strong enzymatic activity in SIF and the dissociation behavior of the INS–SOS complex. The HIP complex, which is prepared at a low pH value, is vulnerable to dissociation in high pH conditions (Noh et al., 2022). A previous report demonstrated that an HIP complex of INS (paired with sodium glycodexoycholate) was rapidly dissociated in pH 7.4 medium (Bashyal et al., 2021). Thus, as a smaller amount of INS was liberated from the INS–SOS complex, a prolonged half-life was seen in SGF. However, only 10% of INS remained after 2 h in SGF, an amount insufficient for oral delivery.

On the other hand, the optimized SMEDDS showed noticeably improved resistance to the enzymes in both SGF and SIF. In SGF, the amount of remaining INS in the optimized SMEDDS was more than 60%, with a *k* value of 0.0034 min^−1^. GI enzymes are mostly hydrophilic; thus, they are incapable of penetrating hydrophobic oil droplets unless the SMEDDS themselves are liable to digestion (Gursoy & Benita, 2004; Zupančič et al., 2017). As pepsin was not able to penetrate the oil phase, INS–SOS was effectively protected in microemulsions, with a half-life of ∼200 min. The degraded INS might be due to INS leakage from the SMEDDS as time passed. In contrast, INS–SOS in the optimized SMEDDS was more unstable in SIF, showing a lower figure of ∼46% remaining INS after 2 h. This finding was attributed to the instability of the optimized SMEDDS in SIF. As lipid droplets are prone to being degraded by both bile salts and lipase in SIF, the INS–SOS complex was released from the microemulsions. The secretion of both bile salts and lipase is promoted by dietary lipids (Vithani et al., 2017). SMEDDSs are mainly composed of lipids; therefore, the secretion of both bile salts and lipase is promoted by the intake of these SMEDDSs (Yin et al., 2009), resulting in digestion of the oil droplets.

In the case of typical small molecules, which have low aqueous solubility, this digestion process is advantageous for increasing oral BA, leading to the generation of mixed micelles, which provide additional solubilizing capacity to the drugs (Weng et al., 2014). For peptide drugs, however, lipid digestion is disadvantageous for oral delivery because they lose their protective vehicles and are thus directly exposed to the digestive enzymes. Nevertheless, the optimized SMEDDS showed superior performance in protecting INS–SOS from enzymatic degradation compared with INS and INS–SOS. In addition, the PM of INS and the SMEDDS was not protected at all in either SGF or SIF, indicating that hydrophilic INS was not suitable for incorporation into the SMEDDS. Thus, for successful development of a SMEDDS formulation containing INS, it is essential to increase the hydrophobicity of INS via HIP complexation. Consequently, undigested INS–SOS in the optimized SMEDDS is expected to increase the oral BA of INS.

### 3.11. *INS leakage from the INS*–*SOS-loaded SMEDDS*

**Figure 7.**
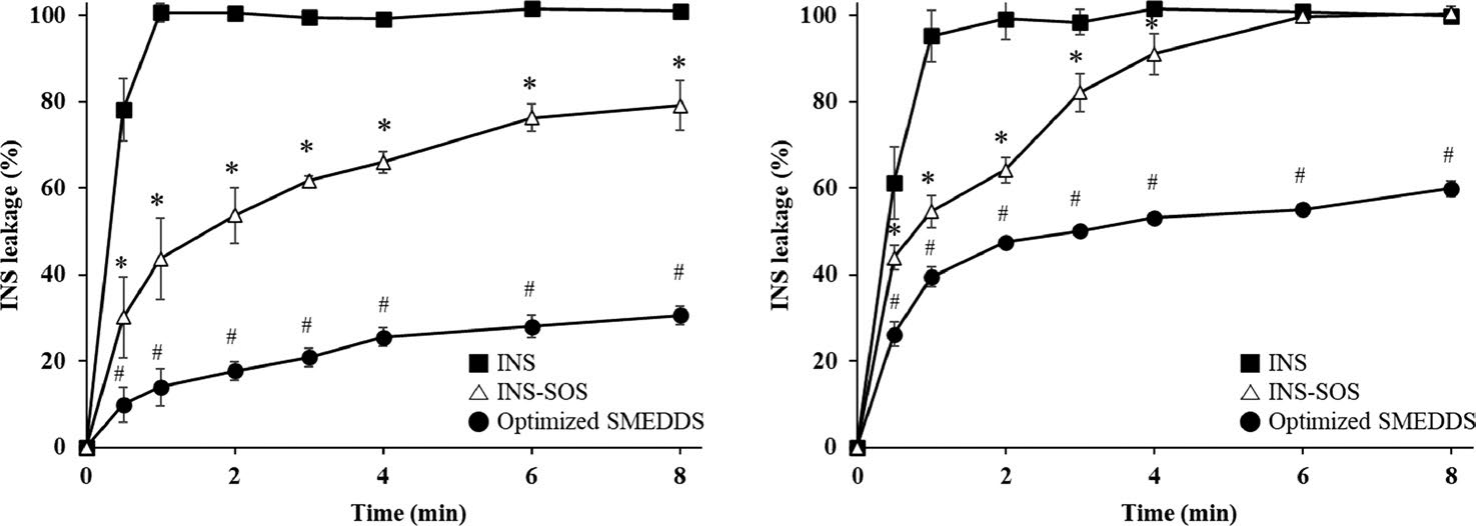
Release profiles of INS, INS–SOS, and the optimized SMEDDS in pH 1.2 medium with 1% Labrasol (left) and pH 6.8 medium (right). Values are presented as mean ± standard deviation (*n* = 3). Significantly different at *p* < .05 resulted from post hoc Tukey’s test: *versus INS; ^#^versus INS and INS–SOS. INS, insulin; SOS, sodium *n*-octadecyl sulfate; SMEDDS, self-microemulsifying drug delivery system.

To determine whether INS remained in the oil droplets following the self-microemulsification of the INS–SOS-loaded SMEDDS, the release behavior of INS across a dialysis membrane in both pH 1.2 medium with 1% Labrasol and pH 6.8 medium was experimentally examined. Figure 7 depicts the release profiles of the INS solution, which served as the control, INS–SOS suspension, and the optimized SMEDDS. As expected, the INS solution rapidly passed through the dialysis membrane in both media. The INS leakage of the INS–SOS complex gradually increased as INS–SOS was either dissolved or dissociated. The significantly slower release profile of the optimized SMEDDS indicates that the ionic complexation of INS with SOS led to its efficient incorporation in the SMEDDS. In addition, the absence of a burst release and the sustained release of INS, which were observed in the case of INS–SOS incorporated into the SMEDDS, could be considered advantageous since they could ensure protection of the protein from degradation until it reaches the epithelium and releases close to the absorption site (Hintzen et al., 2014).

### 3.12. In vivo pharmacodynamics

**Figure 8.**
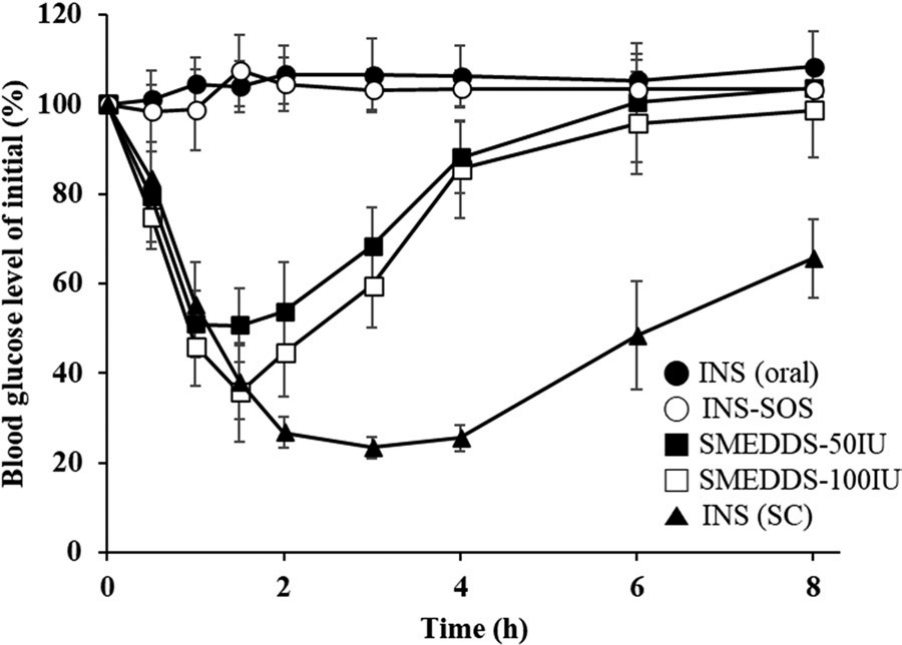
Blood glucose levels in diabetic rats after oral administration of the INS solution (50 IU/kg), INS–SOS solution (50 IU/kg), the optimized SMEDDS at 50 IU/kg (SMEDDS-50IU), and the optimized SMEDDS at 100 IU/kg (SMEDDS-100IU) and after SC administration of the INS solution (5 IU/kg). Values are presented as the mean ± standard deviation. INS, insulin; SOS, sodium *n*-octadecyl sulfate, SMEDDS, self-microemulsifying drug delivery system; SC, subcutaneous.

The pharmacodynamic effect of various INS-loaded formulations was evaluated in diabetic rats following oral administration of a dose of either 50 IU/kg or 100 IU/kg. This was then compared with SC injection of regular INS at a dose of 5 IU/kg. The changes in blood glucose levels (% of initial values) versus time are depicted in Figure 8. SC injection of INS provoked the greatest decrease in blood glucose level, and this effect continued over the whole experimental period. Furthermore, the antidiabetic effects of orally delivered INS and INS–SOS were found to be negligible. On the other hand, the optimized SMEDDS rapidly reduced blood glucose level for the first 2 h, after which the blood glucose level slowly recovered for 6 h. The SMEDDSs with two different doses showed similar patterns; however, the glucose-lowering effect of SMEDDS-100IU was higher than that of SMEDDS-50IU. This rapidly decreasing pattern, as reported in previous literature (Li et al., 2014; Liu et al., 2019), might be caused not only by the protective effect of the optimized SMEDDS against GI enzymes but also by the increased permeation through the GI tract due to the nanosized emulsions (Xia et al., 2021).

The pharmacodynamic parameters of different samples are summarized in Table 6. As expected, orally administered INS and INS–SOS showed little to no hypoglycemic effect, with very low or negative values of AAC during 0–8 h (AAC_0–8h_). These results were attributed to the extreme instability of both INS and INS–SOS in the presence of GI enzymes without an appropriate nanocarrier. In contrast, the AAC_0–8h_ values of SMEDDS-50IU and SMEDDS-100IU were remarkably increased, resulting in AAC_0–8h_ values of approximately 141% and 187%·h, respectively. In addition, the minimum blood glucose level (% of initial) [BGL_min_] values of SMEDDS-50IU and SMEDDS-100IU were 44.90% and 34.67%, respectively, resulting in PA values of 3.23% and 2.13%, respectively. Typically, the SMEDDS formulations were found to enhance the intestinal absorption of low-BA drugs by solubilizing the drugs and facilitating intestinal permeation (Sachs-Barrable et al., 2007; Kale & Patravale, 2008). Although the surface of the SMEDDS was not fabricated, the SMEDDS could also enhance the permeability through mucus, which is prevalently distributed throughout the intestines (Karamanidou et al., 2015). In addition, Menzel et al. (2018) demonstrated the high solubilizing capacity of SMEDDSs for the HIP complex, postulating that ∼85% of exenatide-sodium docusate complex would remain in the SMEDDS droplets prior to the epithelium being reached. Likewise, INS–SOS was effectively solubilized in the oil droplets, ensuring that improved glucose-lowering effects could be obtained.

**Table 6.** Pharmacodynamic parameters of INS in a diabetic rat model following oral administration of INS (50 IU/kg), INS-SOS (50 IU/kg), the optimized SMEDDS at 50 IU/kg (SMEDDS-50IU), the optimized SMEDDS at 100 IU/kg (SMEDDS-100IU), and SC injection of INS solution (5 IU/kg).

Parameter	INS (SC)	INS (oral)	INS–SOS	SMEDDS-50IU	SMEDDS-100IU
Dose (IU/kg)	5	50	50	50	100
AAC_0–8h_ (%·h)	439.29 ± 30.00	–44.47 ± 38.33	–23.84 ± 33.05	141.73 ± 50.67	187.07 ± 51.60
BGL_min_ (%)	22.78 ± 1.92	97.92 ± 1.23	93.60 ± 3.17	44.90 ± 6.39	34.67 ± 10.01
*T*_min_ (h)	3.20 ± 0.84	2.75 ± 0.25	1.08 ± 0.74	1.50 ± 0.35	1.40 ± 0.22
PA (%)	–	–1.01	–0.54	3.23	2.13

Data are expressed as mean ± standard deviation (*n* = 5–7). INS, insulin; SMEDDS, self-microemulsifying drug delivery system; SC, subcutaneous; AAC_0–8h_: area above the blood glucose level curve (% of initial) during 0–8 h; BGL_min_: minimum blood glucose level (% of initial levels); *T*_min_: time to reach BGL_min_; and PA: relative pharmacological availability versus SC injection of INS.

One-way independent groups ANOVA was performed to assess whether different formulations influenced the oral absorption of INS. This analysis yielded a significant effect of treatment on both the AAC_0–8h_ [*F*(4,22) = 120.77, *p* < .001] and BGL_min_ values [*F*(4,22) = 231.94, *p* < .001], suggesting that the extent of absorption of INS differed by administered formulation (Table 7). Furthermore, post hoc Tukey’s test showed that neither the AAC_0–8h_ (mean (*M*) = −23.84) nor the BGL_min_ (*M* = 93.60) values of INS–SOS were significantly different from those of INS (oral) (Supplementary Table S4). In comparison, the SMEDDS formulations significantly increased the oral absorption of INS. The AAC_0–8h_ values of SMEDDS-50IU (*M* = 141.73) and SMEDDS-100IU (*M* = 187.07) were significantly different from those of the other groups, except for the INS (SC) group. In addition, the BGL_min_ values of SMEDDS-50IU (*M* = 44.90) and SMEDDS-100IU (*M* = 34.67) were significantly different from those of the INS (oral) and INS–SOS groups. This indicated that the oral absorption of INS was significantly enhanced by the SMEDDS formulation.

**Table 7.** Analysis of variance results of pharmacodynamics parameters.

Parameter	DF	Sum of square	Mean square	*F* value	*p* value
AAC_0–8h_					
Administration	4	819,692	204,923	120.77	<.001
Residual	22	37328	1697	–	–
BGL_min_					
Administration	4	26,613.8	6,653.44	231.94	<.001
Residual	22	631.1	28.69	–	–

AAC_0–8h_: area above the blood glucose level curve (% of initial) during 0–8 h; BGL_min_: minimum blood glucose level (% of initial levels); DF, degrees of freedom.

Furthermore, SMEDDS-100IU had greater AAC_0–8h_ and BGL_min_ values than those of SMEDDS-50IU, a finding attributed to SMEDDS-100IU being a higher dose than SMEDDS-50IU. In particular, post hoc Tukey’s test demonstrated that the BGL_min_ value of SMEDDS-100IU was significantly different from that of SMEDDS-50IU. However, SMEDDS-50IU had a greater PA value than that of SMEDDS-100IU. This indicated that an excess amount of the SMEDDS might not have been absorbed, indicating that the hypoglycemic effect did not increase proportionally with the dose. This result was comparable with the findings of earlier reports in which orally administered INS-loaded LBFs did not show dose dependency (Sarmento et al., 2007; Xiong et al., 2007). Moreover, a reduction in the total amount of surfactant used is recommended due to the potential for an excess amount of surfactant to induce undesirable GI irritation (Lee et al., 2015). Given this, despite SMEDDS-100IU having a higher AAC_0–8h_ value than SMEDDS-50IU, the dose of 50 IU/kg could be regarded as the suitable dose for the optimized SMEDDS formulation. Therefore, additional approaches such as surface modification and solidification might be needed to obtain the desirable hypoglycemic effect.

## 4. Conclusion

An INS-loaded SMEDDS formulation was successfully optimized using a D-optimal mixture design. Three independent variables—*X*_1_ (Capmul MCM), *X*_2_ (Labrasol), and *X*_3_ (Tetraglycol)—were selected, resulting in droplet sizes (115.2 nm), INS stability (46.75%), and INS leakage (17.67%) with sufficiently low prediction error percentages (<10%). Compared with INS and INS–SOS, the optimized SMEDDS displayed considerable resistance to GI enzymes and diminished INS leakage from the oil droplets. The optimized SMEDDS resulted in PAs of 3.23% (50 IU/kg) and 2.13% (100 IU/kg) in diabetic rats, suggesting the superiority of SMEDDS-50IU over SMEDDS-100IU. Thus, the optimized SMEDDS shows potential for effective delivery of peptide drugs with improved oral absorption. However, as only a rodent model was adopted for this study, further examinations on human pharmacokinetics are necessary for clinical application.

## Supplementary Material

Supplemental Material

## References

[CIT0001] Amaral M, Martins AS, Catarino J, et al. (2020). How can biomolecules improve mucoadhesion of oral insulin? A comprehensive insight using ex-vivo, in silico, and in vivo models. Biomolecules 10:675. 10.3390/biom10050675 PMC7277740 32349416

[CIT0002] Ashar A, Bhatti IA, Siddique T, et al. (2021). Integrated hydrothermal assisted green synthesis of ZnO nano discs and their water purification efficiency together with antimicrobial activity. J Mater Res Technol 15:6901–17.

[CIT0003] Bally L, Thabit H, Hovorka R. (2017). Finding the right route for insulin delivery–an overview of implantable pump therapy. Expert Opin Drug Deliv 14:1103–11. 27911116 10.1080/17425247.2017.1267138 PMC5581917

[CIT0004] Ban C, Jo M, Lim S, Choi YJ. (2018). Control of the gastrointestinal digestion of solid lipid nanoparticles using PEGylated emulsifiers. Food Chem 239:442–52. 28873589 10.1016/j.foodchem.2017.06.137

[CIT0005] Bashyal S, Seo J-E, Choi YW, Lee S. (2021). Bile acid transporter-mediated oral absorption of insulin via hydrophobic ion-pairing approach. J Control Release 338:644–61. 34481926 10.1016/j.jconrel.2021.08.060

[CIT0006] Chamieh J, Tarrat AD, Doudou C, et al. (2019). Peptide release from SEDDS containing hydrophobic ion pair therapeutic peptides measured by Taylor dispersion analysis. Int J Pharm 559:228–34. 30703502 10.1016/j.ijpharm.2019.01.039

[CIT0007] Cho HJ, Lee DW, Marasini N, et al. (2013). Optimization of self-microemulsifying drug delivery system for telmisartan using Box–Behnken design and desirability function. J Pharm Pharmacol 65:1440–50. 24028611 10.1111/jphp.12115

[CIT0008] Coronado M, Segadães AM, Andrés A. (2014). Combining mixture design of experiments with phase diagrams in the evaluation of structural ceramics containing foundry by-products. Appl Clay Sci 101:390–400.

[CIT0009] Cryan S-A. (2005). Carrier-based strategies for targeting protein and peptide drugs to the lungs. Aaps J 7:E20–E41. 16146340 10.1208/aapsj070104 PMC2751494

[CIT0010] Dai W-G, Dong LC. (2007). Characterization of physiochemical and biological properties of an insulin/lauryl sulfate complex formed by hydrophobic ion pairing. Int J Pharm 336:58–66. 17174492 10.1016/j.ijpharm.2006.11.035

[CIT0011] Damgé C, Maincent P, Ubrich N. (2007). Oral delivery of insulin associated to polymeric nanoparticles in diabetic rats. J Control Release 117:163–70. 17141909 10.1016/j.jconrel.2006.10.023

[CIT0012] Dhaval M, Vaghela P, Patel K, et al. (2022). Lipid-based emulsion drug delivery systems—a comprehensive review. Drug Deliv Transl Res 12:1616–39. 34609731 10.1007/s13346-021-01071-9

[CIT0013] Fernandez S, Jannin V, Rodier J-D, et al. (2007). Comparative study on digestive lipase activities on the self emulsifying excipient Labrasol®, medium chain glycerides and PEG esters. Biochim Biophys Acta Mol Cell Biol Lipids 1771:633–40. 10.1016/j.bbalip.2007.02.009 17418634

[CIT0014] Goo YT, Sa C-K, Kim MS, et al. (2022). Enhanced dissolution and bioavailability of revaprazan using self-nanoemulsifying drug delivery system. Pharm Dev Technol 27:414–24. 35467467 10.1080/10837450.2022.2070644

[CIT0015] Griesser J, Hetényi G, Moser M, et al. (2017). Hydrophobic ion pairing: Key to highly payloaded self-emulsifying peptide drug delivery systems. Int J Pharm 520:267–74. 28188875 10.1016/j.ijpharm.2017.02.019

[CIT0016] Gursoy RN, Benita S. (2004). Self-emulsifying drug delivery systems (SEDDS) for improved oral delivery of lipophilic drugs. Biomed Pharmacother 58:173–82. 15082340 10.1016/j.biopha.2004.02.001

[CIT0017] Haddadzadegan S, Dorkoosh F, Bernkop-Schnurch A. (2022). Oral delivery of therapeutic peptides and proteins: Technology landscape of lipid-based nanocarriers. Adv Drug Deliv Rev 182:114097. 34999121 10.1016/j.addr.2021.114097

[CIT0018] Hintzen F, Perera G, Hauptstein S, et al. (2014). In vivo evaluation of an oral self-microemulsifying drug delivery system (SMEDDS) for leuprorelin. Int J Pharm 472:20–6. 24879935 10.1016/j.ijpharm.2014.05.047

[CIT0019] Kale AA, Patravale VB. (2008). Design and evaluation of self-emulsifying drug delivery systems (SEDDS) of nimodipine. AAPS PharmSciTech 9:191–6. 18446481 10.1208/s12249-008-9037-9 PMC2976914

[CIT0020] Karamanidou T, Karidi K, Bourganis V, et al. (2015). Effective incorporation of insulin in mucus permeating self-nanoemulsifying drug delivery systems. Eur J Pharm Biopharm 97:223–9. 25933940 10.1016/j.ejpb.2015.04.013

[CIT0021] Lee DH, Yeom DW, Song YS, et al. (2015). Improved oral absorption of dutasteride via Soluplus®-based supersaturable self-emulsifying drug delivery system (S-SEDDS). Int J Pharm 478:341–7. 25437113 10.1016/j.ijpharm.2014.11.060

[CIT0022] Lee DW, Marasini N, Poudel BK, et al. (2014). Application of Box–Behnken design in the preparation and optimization of fenofibrate-loaded self-microemulsifying drug delivery system (SMEDDS). J Microencapsul 31:31–40. 23834315 10.3109/02652048.2013.805837

[CIT0023] Li P, Tan A, Prestidge CA, et al. (2014). Self-nanoemulsifying drug delivery systems for oral insulin delivery: in vitro and in vivo evaluations of enteric coating and drug loading. Int J Pharm 477:390–8. 25455781 10.1016/j.ijpharm.2014.10.039

[CIT0024] Liao H, Gao Y, Lian C, et al. (2019). Oral absorption and lymphatic transport of baicalein following drug–phospholipid complex incorporation in self-microemulsifying drug delivery systems. Int J Nanomed 14:7291–306. 10.2147/IJN.S214883 PMC6735633 31564878

[CIT0025] Liu J, Werner U, Funke M, et al. (2019). SEDDS for intestinal absorption of insulin: Application of Caco-2 and Caco-2/HT29 co-culture monolayers and intra-jejunal instillation in rats. Int J Pharm 560:377–84. 30790612 10.1016/j.ijpharm.2019.02.014

[CIT0026] Mahmood A, Bernkop-Schnürch A. (2019). SEDDS: A game changing approach for the oral administration of hydrophilic macromolecular drugs. Adv Drug Deliv Rev 142:91–101. 29981355 10.1016/j.addr.2018.07.001

[CIT0027] Menzel C, Holzeisen T, Laffleur F, et al. (2018). In vivo evaluation of an oral self-emulsifying drug delivery system (SEDDS) for exenatide. J Controll Release 277:165–72. 10.1016/j.jconrel.2018.03.018 29574041

[CIT0028] Mura P, Furlanetto S, Cirri M, et al. (2005). Optimization of glibenclamide tablet composition through the combined use of differential scanning calorimetry and D-optimal mixture experimental design. J Pharm Biomed Anal 37:65–71. 15664744 10.1016/j.jpba.2004.09.047

[CIT0029] Nazir I, Asim MH, Dizdarević A, Bernkop-Schnürch A. (2019). Self-emulsifying drug delivery systems: Impact of stability of hydrophobic ion pairs on drug release. Int J Pharm 561:197–205. 30836151 10.1016/j.ijpharm.2019.03.001

[CIT0030] Noh G, Keum T, Bashyal S, et al. (2022). Recent progress in hydrophobic ion-pairing and lipid-based drug delivery systems for enhanced oral delivery of biopharmaceuticals. J Pharm Investig 52:75–93.

[CIT0031] Park SY, Jin CH, Goo YT, et al. (2021). Supersaturable self-microemulsifying drug delivery system enhances dissolution and bioavailability of telmisartan. Pharm Dev Technol 26:60–8. 33032496 10.1080/10837450.2020.1834580

[CIT0032] Patel MH, Sawant KK. (2019). Self microemulsifying drug delivery system of lurasidone hydrochloride for enhanced oral bioavailability by lymphatic targeting: in vitro, Caco-2 cell line and in vivo evaluation. Eur J Pharm Sci 138:105027. 31377133 10.1016/j.ejps.2019.105027

[CIT0033] Peres DDA, Ariede MB, Candido TM, et al. (2017). Quality by design (QbD), Process Analytical Technology (PAT), and design of experiment applied to the development of multifunctional sunscreens. Drug Dev Ind Pharm 43:246–56. 27627681 10.1080/03639045.2016.1236809

[CIT0034] Pillai O, Panchagnula R. (2001). Insulin therapies – past, present and future. Drug Discov Today 6:1056–61. 11590034 10.1016/s1359-6446(01)01962-6

[CIT0035] Quarta E, Chierici V, Flammini L, et al. (2020). Excipient-free pulmonary insulin dry powder: Pharmacokinetic and pharmacodynamics profiles in rats. J Control Release 323:412–20. 32325175 10.1016/j.jconrel.2020.04.015

[CIT0036] Ristroph KD, Prud’homme RK. (2019). Hydrophobic ion pairing: encapsulating small molecules, peptides, and proteins into nanocarriers. Nanoscale Adv 1:4207–37. 33442667 10.1039/c9na00308h PMC7771517

[CIT0037] Sachs-Barrable K, Thamboo A, Lee SD, Wasan KM. (2007). Lipid excipients Peceol and Gelucire 44/14 decrease P-glycoprotein mediated efflux of rhodamine 123 partially due to modifying P-glycoprotein protein expression within Caco-2 cells. J Pharm Pharm Sci 10:319–31. 17727795

[CIT0038] Sarmento B, Ribeiro A, Veiga F, et al. (2007). Oral bioavailability of insulin contained in polysaccharide nanoparticles. Biomacromolecules 8:3054–60. 17877397 10.1021/bm0703923

[CIT0039] Shahzadi I, Nazir I, Phan TNQ, Bernkop-Schnürch A. (2020). About the impact of superassociation of hydrophobic ion pairs on membrane permeability. Eur J Pharm Biopharm 151:1–8. 32268189 10.1016/j.ejpb.2020.03.016

[CIT0040] Shrestha N, Araújo F, Shahbazi MA, et al. (2016). Thiolation and cell-penetrating peptide surface functionalization of porous silicon nanoparticles for oral delivery of insulin. Adv Funct Mater 26:3405–16.

[CIT0041] Son HY, Chae BR, Choi JY, et al. (2018). Optimization of self-microemulsifying drug delivery system for phospholipid complex of telmisartan using D-optimal mixture design. PLoS One 13:e0208339. 30517187 10.1371/journal.pone.0208339 PMC6281252

[CIT0042] Sun S, Cui F, Kawashima Y, et al. (2008). A novel insulin-sodium oleate complex for oral administration: preparation, characterization and in vivo evaluation. J Drug Deliv Sci Technol 18:239–43.

[CIT0043] Sun S, Liang N, Kawashima Y, et al. (2011). Hydrophobic ion pairing of an insulin-sodium deoxycholate complex for oral delivery of insulin. Int J Nanomed 6:3049–56. 10.2147/IJN.S26450 PMC3230571 22162661

[CIT0044] Sun S, Liang N, Yamamoto H, et al. (2015). pH-sensitive poly (lactide-co-glycolide) nanoparticle composite microcapsules for oral delivery of insulin. Int J Nanomed 10:3489–98. 10.2147/IJN.S81715 PMC4435433 25999713

[CIT0045] Suram D, Veerabrahma K. (2022). Design and development of solid SMEDDS and liquisolid formulations of lovastatin, for improved drug dissolution and in vivo effects – a pharmacokinetic and pharmacodynamic assessment. AAPS PharmSciTech 23:123. 35460060 10.1208/s12249-022-02272-2

[CIT0046] Tang J, Bai Y, Chen X. (2021). An investigation of inorganic fillers on rheological properties and tensile strength of epoxy repair coating using mixture design method. Constr Build Mater 307:124866.

[CIT0047] Varanda C, Portugal I, Ribeiro J, et al. (2017). Optimization of bitumen formulations using mixture design of experiments (MDOE). Constr Build Mater 156:611–20.

[CIT0048] Verma S, Goand UK, Husain A, et al. (2021). Challenges of peptide and protein drug delivery by oral route: current strategies to improve the bioavailability. Drug Dev Res 82:927–44. 33988872 10.1002/ddr.21832

[CIT0049] Vithani K, Hawley A, Jannin V, et al. (2017). Inclusion of digestible surfactants in solid SMEDDS formulation removes lag time and influences the formation of structured particles during digestion. Aaps J 19:754–64. 28116678 10.1208/s12248-016-0036-6

[CIT0050] Weng T, Qi J, Lu Y, et al. (2014). The role of lipid-based nano delivery systems on oral bioavailability enhancement of fenofibrate, a BCS II drug: comparison with fast-release formulations. J Nanobiotechnol 12:39. 10.1186/s12951-014-0039-3 PMC4180958 25248304

[CIT0051] Wibel R, Friedl JD, Zaichik S, Bernkop-Schnürch A. (2020). Hydrophobic ion pairing (HIP) of (poly) peptide drugs: benefits and drawbacks of different preparation methods. Eur J Pharm Biopharm 151:73–80. 32289492 10.1016/j.ejpb.2020.04.004

[CIT0052] Xia F, Chen Z, Zhu Q, et al. (2021). Gastrointestinal lipolysis and trans-epithelial transport of SMEDDS via oral route. Acta Pharm Sin B 11:1010–20. 33996413 10.1016/j.apsb.2021.03.006 PMC8105768

[CIT0053] Xiong XY, Li YP, Li ZL, et al. (2007). Vesicles from pluronic/poly (lactic acid) block copolymers as new carriers for oral insulin delivery. J Controll Release 120:11–7. 10.1016/j.jconrel.2007.04.004 17509718

[CIT0054] Yao J, Lu Y, Zhou JP. (2008). Preparation of nobiletin in self-microemulsifying systems and its intestinal permeability in rats. J Pharm Pharm Sci 11:22–9. 10.18433/j3ms3m 18801304

[CIT0055] Yeom DW, Song YS, Kim SR, et al. (2015). Development and optimization of a self-microemulsifying drug delivery system for ator vastatin calcium by using D-optimal mixture design. Int J Nanomedicine 10:3865–77. 26089663 10.2147/IJN.S83520 PMC4462857

[CIT0056] Yin Y-M, Cui F-D, Mu C-F, et al. (2009). Docetaxel microemulsion for enhanced oral bioavailability: preparation and in vitro and in vivo evaluation. J Controll Release 140:86–94. 10.1016/j.jconrel.2009.08.015 19709639

[CIT0057] Zhang J, Wen X, Dai Y, Xia Y. (2019). Mechanistic studies on the absorption enhancement of a self-nanoemulsifying drug delivery system loaded with norisoboldine-phospholipid complex. Int J Nanomedicine 14:7095–106. 31564867 10.2147/IJN.S211905 PMC6730610

[CIT0058] Zhang N, Ping QN, Huang GH, Xu WF. (2005). Investigation of lectin-modified insulin liposomes as carriers for oral administration. Int J Pharm 294:247–59. 15814248 10.1016/j.ijpharm.2005.01.018

[CIT0059] Zhang Q, He N, Zhang L, et al. (2012). The in vitro and in vivo study on self-nanoemulsifying drug delivery system (SNEDDS) based on insulin-phospholipid complex. J Biomed Nanotechnol 8:90–7. 22515097 10.1166/jbn.2012.1371

[CIT0060] Zhang Q, Wang X, Xue H, et al. (2021). Determination and comparison of the solubility, oil–water partition coefficient, intestinal absorption, and biliary excretion of carvedilol enantiomers. AAPS PharmSciTech 22:43. 33426619 10.1208/s12249-020-01906-7

[CIT0061] Zhang T, Tang JZ, Fei X, et al. (2021). Can nanoparticles and nano–protein interactions bring a bright future for insulin delivery? Acta Pharm Sin B 11:651–67. 33777673 10.1016/j.apsb.2020.08.016 PMC7982494

[CIT0062] Zhao X, Zu Y, Zu S, et al. (2010). Insulin nanoparticles for transdermal delivery: preparation and physicochemical characterization and in vitro evaluation. Drug Dev Ind Pharm 36:1177–85. 20367030 10.3109/03639041003695089

[CIT0063] Zupančič O, Rohrer J, Thanh Lam H, et al. (2017). Development and in vitro characterization of self-emulsifying drug delivery system (SEDDS) for oral opioid peptide delivery. Drug Dev Ind Pharm 43:1694–702. 28589736 10.1080/03639045.2017.1338722

