# Supplementary material for: Enhanced oral absorption of insulin: hydrophobic ion pairing and a self-microemulsifying drug delivery system using a D-optimal mixture design
Source: Drug Deliv. 2022 Sep 1;29(1):2831–45. doi: 10.1080/10717544.2022.2118399 (PMC9448375; doi:10.1080/10717544.2022.2118399)

**Table S1.** Degradation kinetics of INS-SOS at different NaCl concentrations

|  | Rate constant (min^-1^) | Half-life (min) |
| --- | --- | --- |
| 0 mM | 0.0244 | 28.41 |
| 100 mM | 0.0313 | 22.15 |
| 150 mM ^a^ | 0.0360 | 19.25 |
| 200 mM | 0.0392 | 17.68 |

^a^ isotonic condition. INS, insulin; SOS, sodium *n*-octadecyl sulfate.

**Table S2.** Combinations of independent variables and experimental responses obtained from runs

| Run | X_1_ (%) | X_2_ (%) | X_3_ (%) | Y_1_ (nm) | Y_2_ (%) | Y_3_ (%) |
| --- | --- | --- | --- | --- | --- | --- |
| 1 | 5.00 | 25.00 | 70.00 | 175.0±0.6 | 27.74±3.97 | 29.37±4.73 |
| 2 | 5.00 | 42.82 | 52.18 | 131.6±1.6 | 51.22±4.84 | 26.30±4.23 |
| 3 | 5.00 | 25.00 | 70.00 | 166.0±2.2 | 25.15±4.91 | 29.86±3.51 |
| 4 | 5.00 | 60.00 | 35.00 | 95.8±1.2 | 55.33±4.13 | 35.77±4.27 |
| 5 | 8.56 | 28.78 | 62.66 | 171.7±0.8 | 30.24±4.15 | 23.71±3.07 |
| 6 | 5.00 | 36.17 | 58.83 | 159.5±1.2 | 49.57±4.16 | 30.61±3.39 |
| 7 | 12.12 | 52.12 | 35.77 | 121.5±1.6 | 48.52±4.81 | 19.54±3.33 |
| 8 | 20.00 | 45.00 | 35.00 | 178.5±0.8 | 35.99±2.56 | 16.69±2.91 |
| 9 | 12.89 | 42.32 | 44.79 | 130.1±0.2 | 41.22±3.11 | 17.53±3.43 |
| 10 | 20.00 | 45.00 | 35.00 | 180.0±1.8 | 34.13±4.55 | 19.68±4.05 |
| 11 | 20.00 | 32.44 | 47.56 | 234.8±1.2 | 33.94±3.49 | 17.02±6.28 |
| 12 | 12.54 | 34.24 | 53.22 | 152.2±0.9 | 40.11±3.80 | 19.14±2.69 |
| 13 | 5.00 | 50.92 | 44.08 | 104.6±1.7 | 57.12±3.15 | 30.51±2.87 |
| 14 | 16.27 | 25.00 | 58.73 | 247.2±3.5 | 33.07±2.86 | 20.51±3.27 |
| 15 | 5.00 | 60.00 | 35.00 | 104.4±0.7 | 51.88±3.15 | 39.29±4.78 |
| 16 | 20.00 | 32.44 | 47.56 | 231.8±1.3 | 33.14±3.41 | 15.58±2.88 |
| 17 | 16.27 | 25.00 | 58.73 | 244.4±2.7 | 31.49±3.88 | 22.81±4.71 |

X_1_: Capmul MCM, X_2_: Labrasol, X_3_: Tetraglycol, Y_1_: droplet size, Y_2_: remaining INS in simulated intestinal fluid at 2 h, Y_3_: INS leakage for 2 h. INS, insulin. Values are presented as the mean ± standard deviation (n = 3).

**Table S3.** Degradation kinetics of INS in different formulations in simulated gastric fluid and simulated intestinal fluid

|  | Simulated gastric fluid | |  | Simulated intestinal fluid | |
| --- | --- | --- | --- | --- | --- |
|  | Rate constant (min^-1^) | Half-life (min) |  | Rate constant (min^-1^) | Half-life (min) |
| INS | 0.1854 | 3.74 |  | 0.1980 | 3.50 |
| INS-SOS | 0.0165 | 42.01 |  | 0.0337 | 20.57 |
| PM of INS and SMEDDS | 0.1575 | 4.40 |  | 0.1776 | 3.90 |
| Optimized SMEDDS | 0.0034 | 203.87 |  | 0.0045 | 154.03 |

INS, insulin; SOS, sodium *n*-octadecyl sulfate; PM, physical mixture; SMEDDS, self-microemulsifying drug delivery system.

**Table S4a**. Post-hoc comparison results of the area above the curve value

|  |  | Mean difference | SE | t-value | P_tukey_ |
| --- | --- | --- | --- | --- | --- |
| INS (SC) | INS (oral) | 483.8 | 24.9 | 19.39 | < 0.001 |
|  | INS-SOS | 463.1 | 24.9 | 18.57 | < 0.001 |
|  | SMEDDS-50IU | 297.6 | 26.1 | 11.42 | < 0.001 |
|  | SMEDDS-100IU | 252.2 | 26.1 | 9.68 | < 0.001 |
| SMEDDS-50IU | INS (oral) | 186.2 | 24.9 | 7.47 | < 0.001 |
|  | INS-SOS | 165.6 | 24.9 | 6.64 | < 0.001 |
|  | SMEDDS-100IU | –45.3 | 26.1 | –1.74 | 0.431 |
| SMEDDS-100IU | INS (oral) | 231.5 | 24.9 | 9.28 | < 0.001 |
|  | INS-SOS | 210.9 | 24.9 | 8.46 | < 0.001 |
| INS-SOS | INS (oral) | 20.6 | 23.8 | 0.87 | 0.906 |

SE, standard error; INS, insulin, SC, subcutaneous, SOS, sodium *n*-octadecyl sulfate; SMEDDS, self-microemulsifying drug delivery system.

**Table S4b.** Post-hoc comparison results of the blood glucose level

|  |  | Mean difference | SE | t-value | P_tukey_ |
| --- | --- | --- | --- | --- | --- |
| INS (SC) | INS (oral) | –75.14 | 3.24 | –23.17 | < 0.001 |
|  | INS-SOS | –70.82 | 3.24 | –21.84 | < 0.001 |
|  | SMEDDS-50IU | –22.12 | 3.39 | –6.53 | < 0.001 |
|  | SMEDDS-100IU | –11.89 | 3.39 | –3.51 | 0.015 |
| SMEDDS-50IU | INS (oral) | –53.02 | 3.24 | –16.35 | < 0.001 |
|  | INS-SOS | –48.70 | 3.24 | –15.02 | < 0.001 |
|  | SMEDDS-100IU | 10.22 | 3.39 | 3.02 | 0.045 |
| SMEDDS-100IU | INS (oral) | –63.25 | 3.24 | –19.50 | < 0.001 |
|  | INS-SOS | –58.93 | 3.24 | –18.17 | < 0.001 |
| INS-SOS | INS (oral) | –4.32 | 3.09 | –1.40 | 0.635 |

SE, standard error; INS, insulin, SC, subcutaneous, SOS, sodium n-octadecyl sulfate; SMEDDS, self-microemulsifying drug delivery system.

**Supplementary Figure Legend**

**Figure S1.** Adequacy of the cubic model after checking the normality of the residuals and outliers of the responses Y_1_, Y_2_, and Y_3_. (A) Normal % probability plot versus internally studentized residuals. (B) Externally studentized residuals plot versus run number. Y_1_: droplet size, Y_2_: INS stability, Y_3_: INS leakage. INS, insulin.

**Figure S1**

**[Goo et al.]**


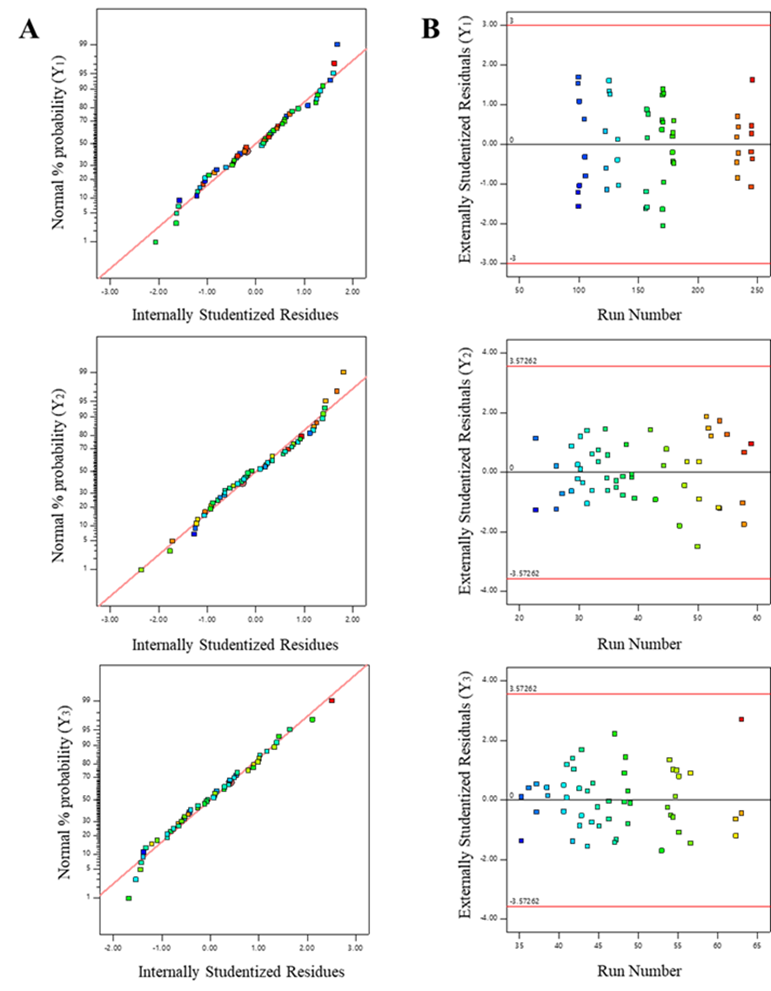

Supplement: Supplemental Material [file IDRD_A_2118399_SM1941.docx]
